# Prolonged treatment with the synthetic glucocorticoid methylprednisolone affects adrenal steroidogenic function and response to inflammatory stress in the rat

**DOI:** 10.1016/j.bbi.2020.03.001

**Published:** 2020-07

**Authors:** Francesca Spiga, Zidong Zhao, Stafford L. Lightman

**Affiliations:** Bristol Medical School, Translational Health Sciences, University of Bristol, Bristol, United Kingdom

**Keywords:** Glucocorticoids, Inflammation, Adrenal gland, Steroidogenesis, HPA axis, Adrenal insufficiency

## Abstract

Synthetic glucocorticoids are widely prescribed for the treatment of numerous inflammatory and autoimmune diseases and they can also affect the way the adrenal gland produces endogenous glucocorticoids. Indeed, patients undergoing synthetic glucocorticoid treatment can develop adrenal insufficiency, a condition characterised by reduced responsiveness of the adrenal to ACTH stimulation or stressors (e.g. surgical or inflammatory stress). To better elucidate the long-term effect of synthetic glucocorticoids treatment and withdrawal on adrenal function, we have investigated the long-term effects of prolonged treatment with methylprednisolone on HPA axis dynamics and on the adrenal steroidogenic pathway, both in basal conditions and in response to an inflammatory stress (lipopolysaccharide, LPS).

We have found that 5-days treatment with methylprednisolone suppresses basal ACTH and corticosterone secretion, as well as corticosterone secretion in response to a high dose of ACTH, and down-regulates key genes in the adrenal steroidogenic pathway, including StAR, MRAP, CYP11a1 and CYP11b1. These effects were paralleled by changes in the adrenal expression of transcription factors regulating steroidogenic gene expression, as well as changes in the expression of adrenal clock genes. Importantly, 5 days after withdrawal of the treatment, ACTH levels are restored, yet basal levels of corticosterone, as well as most of the key steroidogenic genes and their regulators, remain down regulated. We also show that, although 5-days treatment with methylprednisolone reduces the corticosterone response to LPS, an increase in intra-adrenal pro-inflammatory cytokine gene expression was observed.

Our data suggests that the steroidogenic pathway is directly affected by synthetic glucocorticoid treatment in the long-term, presumably via a mechanism involving activation of the glucocorticoid receptor. Furthermore, our data suggests a pro-inflammatory effect of synthetic glucocorticoids treatment in the adrenal gland.

## Highlights

• Prolonged MPRED treatment disrupts basal corticosterone ultradian rhythm.
• Prolonged MPRED treatment decreases adrenal response to ACTH.
• These effects are associated with decreased expression of steroidogenic proteins.
• Prolonged MPRED treatment increases adrenal pro-inflammatory cytokine expression.

## 1 Introduction

Glucocorticoids hormones (GCs) are the final product resulting from activation of the hypothalamic pituitary adrenal (HPA) axis in response to circadian cues and to stress. They are synthesized by the adrenal gland in response to stimulation by adrenocorticotropic hormone (ACTH), which is secreted by the anterior pituitary in response to corticotrophin-releasing hormone and arginine vasopressin released from hypothalamic paraventricular neurons. Glucocorticoids regulate many physiological functions, including metabolism, cardiovascular tone, reproduction, mood and cognition, and the immune system. In addition, GCs regulate their own production through negative feedback on ACTH and CRH secretion (Spiga et al., 2014).

Because of their effect on the immune system, synthetic GC (SGC) therapy is widely prescribed for the treatment of inflammatory and autoimmune diseases. However, the therapeutic actions of SGCs are accompanied by several adverse side effects, which are more frequent following high doses and long-term treatments. Importantly, patients undergoing SGC treatment can also develop adrenal insufficiency following prolonged treatment, due to chronic glucocorticoid-induced inhibition of the HPA axis. This condition is characterised by reduced responsiveness of the adrenal gland to ACTH stimulation, and adrenal crisis/shock can occur in response to acute physiological stress (e.g. surgical or inflammatory stress). Importantly, there is a correlation between duration of SGC treatment and the reduction in adrenal responsiveness, as well as its recovery to normal function. Indeed, some studies have shown that it can take more than a year for a suppressed adrenal to recover after long-term SGC treatment, whereas very rapid recovery within a day or so has been observed in patients that have only been treated for 1–2 weeks (Paragliola et al., 2017).

Previous studies have concentrated on long-term inhibitory effects of SGC treatment at the level of the hypothalamus and pituitary but have tended to ignore the powerful direct effects that these steroids may have at the level of the adrenal itself. Since ACTH is necessary for maintenance of steroidogenic activity, it is often assumed that SCG mediated adrenal insufficiency is solely due to suppression of ACTH. However, our previous data in the rat shows that the SGC methylprednisolone (MPRED) not only suppresses ACTH and corticosterone secretion, but also down-regulates key genes within the adrenal steroidogenic pathway. The same study also showed that pulsatile administration of ACTH could prevent the effects of SGC treatment on both corticosterone secretion and steroidogenic gene expression (Spiga et al., 2011). Furthermore a direct effect of glucocorticoids on the expression of some key steroidogenic genes has already been described (Peron et al., 1960, Carsia and Malamed, 1979, Loose et al., 1980, Gummow et al., 2006). More recently, using *in vivo* experiments in the rat and mathematical modelling, we were able to show that activation of the glucocorticoid receptor (GR) in the adrenal gland can indeed down regulate the steroidogenic response to ACTH (Walker et al., 2015, Spiga et al., 2017) and a more recent study has reported the presence of a negative glucocorticoid responsive element within the promoter of steroidogenic genes (Wang et al., 2019). Therefore, we hypothesised that glucocorticoid-induced long-term changes in the adrenal gland steroidogenic pathways may also be responsible for adrenal suppression.

We have previously shown that 5-days continuous treatment with MPRED in the drinking water suppresses basal secretion of the endogenous GC corticosterone in the rat, and this effect was associated with decreased expression of key steroidogenic gene including StAR, CYP11a1 and MRAP (Spiga et al., 2011). Although this study clearly showed a link between decreased corticosterone secretion and changes in steroidogenic gene expression, a limitation of our previous work was the small numbers of steroidogenic genes measured, as well as the lack of investigation on other factors known to regulate steroidogenic pathways. Indeed, we have recently shown that GC production in the adrenal gland is governed by complex dynamic interactions between components of the steroidogenic regulatory network (Spiga et al., 2017). Therefore, in the present study we made our investigation more comprehensive by including the expression of a larger number of genes within the adrenal steroidogenic pathway, and other genes that are known to be involved in the regulation of glucocorticoid synthesis, including transcription factors and nuclear receptors, as well as clock genes and inflammatory modulators. This approach allowed us to expand on our previous work and to show that long-term treatment with SGCs can indeed down-regulate adrenal steroidogenic activity both directly by regulating the expression of specific steroidogenic genes, and indirectly, by affecting other signalling pathways. Furthermore, we have investigated the long-term effects of prolonged MPRED treatment on the hormonal and adrenal response to endotoxin, and we have found that there is an increase in the intra-adrenal cytokines IL-1β and TNFα in parallel to the decreased corticosterone response to inflammation. Thus, our data provide evidence of a pro-inflammatory effect of SGCs in the adrenal gland that, in the long-term, may contribute to further aggravation of the already-disrupted adrenal steroidogenic activity.

## 2 Material and methods

### 2.1 Animals

All experiments were conducted on adult male Sprague–Dawley rats (Harlan Laboratories, Inc., Blackthorn, UK) weighting 200–220 g at the time of arrival. Animals were given a 1-week acclimatization period prior to the start of the experiments, they were maintained under a 14 h light, 10 h dark schedule (lights on at 0500 h) and housed four per cage with ad libitum access to food and water. All animal procedures were approved by the University of Bristol Ethical Review, comply with the ARRIVE guidelines and were carried out in accordance with the U.K. Animals (Scientific Procedures) Act, 1986.

### 2.2 Methylprednisolone treatment

Rats were assigned to each treatment group randomly. Rats were either left untreated (control group, Ctrl), treated with methylprednisolone (MP; methylprednisolone sodium succinate, Solu-Medrone, Upjohn Pharmaceuticals, UK) in the drinking water (1 g/L) for 5 days (MP treatment group, MPT), or treated with MP for 5 days and then left to recover for 5 days (MP recovery group, MPR). Because high doses of synthetic glucocorticoids have been shown to decrease body weight in the rat, the efficacy of the MP treatment, and withdraw from it, was monitored through the treatment by assessing the rats body weight (Suppl. Fig. 1).

### 2.3 Experiments

*Experiment 1* was designed to determine the effect of MP treatment and recovery on basal circadian and ultradian rhythms of corticosterone and the adrenal response to ACTH. On day 5 of treatment rats were anesthetized using isoflurane, and an indwelling catheter was inserted in the right jugular vein as previously described (Spiga et al., 2011). On day 10 of treatment rats were connected to an automated blood sampling system as previously described (Spiga et al., 2011), blood samples were collected every 10 min from 07:00 of for 24 h at a dilution of 1:5 in heparinized saline and each sample from the automated system contained no more than 40 μl of whole blood. On day 11 at 7:00 rats were injected with ACTH (16 ng/rat, i.v.; Synacthen, Alliance Pharma, Cheltenham, United Kingdom) and samples were collected every 10 min for 60 min after the injection. Corticosterone levels were analysed using radioimmunoassay (RIA) as previously shown (Spiga et al., 2011). *Experiment 2* was designed to determine the effect of MP treatment and recovery on gene and protein expression in the hypothalamus, anterior pituitary and adrenal gland during the nadir (9 AM) and the peak (5 PM) of HPA axis activity. *Experiment 3* was designed to determine the effect of MP treatment and recovery on the adrenal response to inflammatory stress. Rats were treated with MP and then injected with lipopolysaccharide (LPS; Escherichia coli, clone 055:B5; 250 μg/kg in 0.1 mL of sterile saline; Sigma, Dorset, United Kingdom).

### 2.4 Tissue collection

At the end of experiment 1, rats were overdosed with 0.2 mL of sodium pentobarbitone (Euthatal, 200 mg/mL; Merial, Harlow, United Kingdom). At the end of experiment 2 and 3 rats were anaesthetised with isoflurane and killed by decapitation, trunk blood was collected in ice-cold tubes containing EDTA (0.5 M; pH 7.4) and Trasylol (Aprotinin, 500,000 KIU/mL, Roche Diagnostics). Plasma was separated by centrifugation and then stored at –80 °C until processed for ACTH and corticosterone measurement. Adrenal glands were collected and the inner zones (comprising the *zona fasciculata* and the *zona reticularis* of the cortex and the adrenal medulla) were separated from the outer zone (containing the *zona glomerulosa* and the *capsula*). Individual inner zones were immediately frozen until processing for isolation of RNA for real-time quantitative PCR (left adrenal) and for protein extraction for Western immunoblotting and corticosterone measurement (right adrenal) as previously described (Spiga et al., 2017). In experiment 2 pituitaries and brains were also collected, and from each rat anterior pituitary and hypothalamus were dissected and immediately frozen until processing for isolation of RNA for real-time quantitative PCR. In experiments 2 and 3, liver was also collected and immediately frozen until processing for isolation of RNA for real-time quantitative PCR.

### 2.5 RNA isolation and RT-qPCR

Total RNA was extracted from the inner zone each right adrenal, and from the liver, anterior pituitary and the hypothalamus using TRIzol reagent (Invitrogen, Hopkinton, MA, USA), followed by purification using RNeasy mini kit reagents, and column DNase digestion (Qiagen, Valencia, CA, USA) to remove genomic DNA contamination. Complementary DNA was reverse transcribed from 1 μg of total RNA using Cloned AMV First-Strand cDNA synthesis kit (Invitrogen). Fast SYBRGreen Master Mix (Applied Biosystems, Foster City, CA, USA) was used for the amplification mixture with each primer at a final concentration of 200 nm and 2 μl of cDNA for a total reaction volume of 25 μl. All primers were purchased from Invitrogen and were designed to span exon/exon boundaries (Suppl. Table S1). PCR reactions were performed on a spectrofluorometric thermal cycler. The expression of each target gene was normalized to actin mRNA as determined in a separate real-time PCR reaction. The absence of RNA detection when the reverse transcription step was omitted indicated the lack of genomic DNA contamination in the RNA samples.

### 2.6 Western immunoblotting

Whole cell lysate from the inner zone of individual left adrenals were prepared using RIPA buffer (Sigma) supplemented with 0.2 mM Na orthovanadate, 2 mM NaF, and Complete Protease Inhibitor (Roche Diagnostics Ltd., Burgess Hill, UK). Protein concentration was quantified by spectrophotometry using the Pierce BCA protein assays, (Thermo Fisher Scientific Inc. Rockford, IL, USA). Aliquots of each sample (10–15 μg of protein) were loaded and separated in a 10% or 4–15% Tris–Glycine gel (BioRad, Hercules, CA, USA), transferred to a PVD membrane (GE Amersham Biosciences, Piscataway, NJ, USA), blocked with 5% non-fat milk or 1% bovine serum albumin (BSA, sigma) in 1 × Tris-buffered saline plus 0.05% Tween 20 (TBST) and incubated overnight with Aliquots of each sample (10–15 μg of protein) were loaded and separated in a 10% or 4–15% Tris–Glycine gel (BioRad, Hercules, CA, USA), transferred to a PVD membrane (GE Amersham Biosciences, Piscataway, NJ, USA), blocked with 5% non-fat milk or 1% bovine serum albumin (BSA, sigma) in 1 × Tris-buffered saline plus 0.05% Tween 20 (TBST) and incubated overnight with antibodies to StAR and HSL (at 1:1000 dilution, Santa Cruz Biotechnologies, Inc., Dallas, TX, US). After washing with TBST, the membranes were incubated with a horseradish peroxidase-conjugated donkey anti-rabbit IgG (at 1:10000 dilution; Santa Cruz Biotechnologies). After StAR an HSL bands exposure, blots were stripped and assayed for GAPDH (at 1:5000 dilution; Cell Signalling Technology, Inc., USA). Protein bands were visualised with Luminata Forte Western HRP substrate (Millipore Corporation, Billerica, MA, USA) using a G BOX (Syngene, Cambridge, UK) and densitometry was determined using Image J (developed at the National Institutes of Health and freely available at: http://rsb.info.nih.gov). Data points for each gene were then normalized relative to the GAPDH band in the respective sample.

### 2.7 Hormone measurement

Adrenal CORT was measured in adrenal whole cell extract prepared for Western blotting and CORT levels were normalized to the total protein content. Corticosterone concentration was measured in diluted whole blood or plasma in samples obtained with the automatic blood sampling system or from the trunk blood, respectively. Total blood, plasma and adrenal CORT was measured by radioimmunoassay (RIA) using a citrate buffer (pH 3.0) to denature the binding globulin as previously described (Spiga et al., 2017). Antiserum was kindly supplied by Professor Gabor Makara (Institute of Experimental Medicine, Budapest, Hungary) and [125I] CORT was purchased from Izotop (Budapest, Hungary). ACTH concentration in plasma samples was measured by RIA using a commercially available assay (MP Biomedicals, Santa Ana, California, USA) in accordance with the manufacturer’s instructions.

### 2.8 Statistics

Sample sizes in each experiment were determined on the basis of pilot studies and previous experience with similar experimental design. Animals were allocated to each experimental group (treatment and time of kill) by simple randomization. Experimenters were blinded to the experimental group at the time of hormones, mRNA and protein measurements. Graph Pad Prism version 7.00 (Graph Pad Software, La Jolla, CA, USA) and SPSS version 24 (IBM Corp., Armonk, NY, USA) were used for data graphing and statistical analysis, respectively. Data are represented as the mean ± SEM, mRNA and protein data are expressed as fold induction of Ctrl AM. Parameters characterizing the pulses of corticosterone were analysed in individual profile using PULSAR algorithm (Merriam and Wachter, 1982) as previously described (Spiga et al., 2011). The overall effect of treatments was analysed using unpaired *t*-test, or One-Way or Two-Way ANOVA followed by Tuckey post-hoc test, as indicated in each figure legend. Details of statistical significances for each experiment are reported in Suppl. Tables 2 and 3. Statistical significance was set at P ≤ 0.05.

## 3 Results

### 3.1 Prolonged treatment with MPRED decreases basal corticosterone secretion and the adrenal response to ACTH

**Fig. 1:**
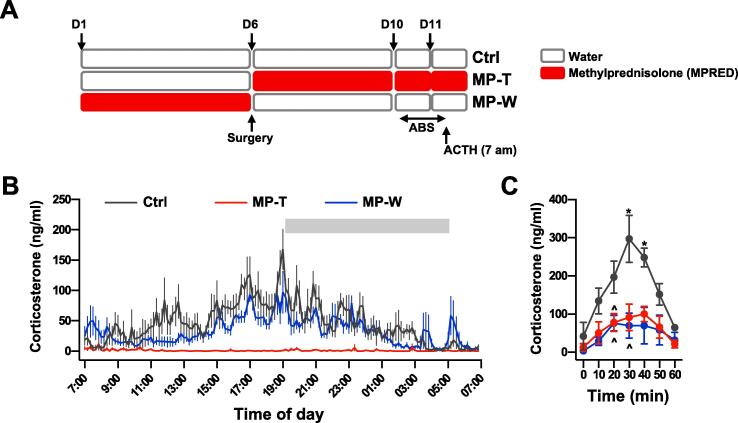
Effect of MPRED treatment and recovery on basal and ACTH induced corticosterone secretion. (A) Schematic representation of the basal ultradian rhythm and ACTH injection experiments using automated blood sampling experiment. (B) Mean ± SEM corticosterone profiles from untreated rats (control group, Ctrl; n = 7), rats treated with methylprednisolone (MPRED) in the drinking water (1 g/L) for 5 days (MPRED treatment group, MP-T; n = 6), and rats treated with MPRED for 5 days and then left to recover for 5 days (MPRED withdraw group, MP-W; n = 7). Individual profiles from rats in each experimental group are reported in Suppl. Fig. 1. Blood samples were collected every 10 min from 07:00 on day 1 for 24 h using an automated blood sampling system. Corticosterone secretion parameters and pulse dynamics characteristics from each individual profile were analysed using the PULSAR algorithm and data are shown in Suppl. Table 2A and B. Gray bar represents the dark period (19:00–05:00). (C) Time course of plasma corticosterone response to ACTH administration (16 ng, i.v.). Data were analysed by repeated measures ANOVA and Tukey post-hoc test. *P < 0.05 vs time 0 (effect of ACTH); ^P < 0.05 vs Ctrl at the same time.

Under basal, unstressed, conditions corticosterone secretion in the rat exhibits ultradian oscillations, characterised by hourly pulses of hormone secretion, and the amplitude of these pulses varies in a circadian manner with larger pulses occurring at the start of the active phase (early-evening in the rat) (Windle et al., 1998, Spiga et al., 2014). We used an automated blood sampling system to assess the effects of MPRED treatment on basal corticosterone circadian and ultradian rhythms, as well as on corticosterone pulse dynamics (Fig. 1A). Analysis of corticosterone secretion in Ctrl rats showed the well-characterised ultradian and circadian patterns (Fig. 1B and Suppl. Fig. 2A), with corticosterone secretion parameters and pulse characteristics, analysed across the 24-h hormone profile using the PULSAR algorithm, that were as previously reported (Seale et al., 2004, Spiga et al., 2011) (Suppl. Table 2). In contrast, and as reported in previous studies (Spiga et al., 2011), 5-days treatment with MPRED suppressed basal corticosterone secretion, as shown by almost undetectable levels of hormone through the 24-h period (MP-T rats; Fig. 1B and Suppl. Fig. 2B). Importantly, 5 days after withdrawing from the treatment basal corticosterone levels appeared to be still significantly lower than the Ctrl group (MP-W group; Fig. 1B and Suppl. Fig. 2C), as shown by a decreased mean, max and 24-h AUC corticosterone levels (Suppl. Table 2). Analysis of corticosterone pulse characteristics with the PULSAR algorithm revealed a decrease in the pulse amplitude and length, as well as a decrease in pulse frequency, and an increase in the inter-pulse interval time and in the pulse length in MP-W rats, compared to Ctrl (Suppl. Table 2). In addition to a decrease in basal corticosterone secretion, we also observed an effect of MPRED treatment on the adrenal response to ACTH (*treatment:* F_(2,97)_ = 25.32; P < 0.0001*;* Fig. 1C)*,* with corticosterone secretion significantly reduced in both MP-T and MP-W group. Interestingly, while basal corticosterone secretion was partially recovered in MP-W rats (Fig. 1B, Suppl. Table 2), adrenal response to ACTH in this group was not different from MP-T group, suggesting that long-term changes in the adrenal gland may occur after MPRED treatment, and persist even after discontinuation of the treatment.

To investigate whether the changes in basal and ACTH-induced corticosterone secretion in MP-T and MP-W rats were due to the effects of MPRED on hypothalamic-pituitary activity, as well as a direct effect on adrenal steroidogenic function, we repeated the MPRED treatment in a different cohort of rats and we collected trunk blood samples, hypothalamus, pituitary and adrenals both in the morning (09:00, AM) and in the evening (19:00, PM), that is at the nadir and at the peak time of corticosterone secretion. Analysis of HPA axis-related genes in the whole hypothalamus and anterior pituitary revealed that hypothalamic CRH mRNA and AVP mRNA, and pituitary POMC mRNA and CRHR1 mRNA levels were unaffected by MPRED treatment (Suppl. Fig. 3A and B). In addition, while the expression of pituitary glucocorticoid and mineralocorticoid receptors (GR and MR, respectively), which mediate the effects of GC-negative feedback, was also unaffected by MPRED treatment (Suppl. Fig. 3C), an increase in GR mRNA in the hypothalamus was found in MP-W rats (*treatment*: F_(2, 34)_ = 4.25; P = 0.024; Suppl. Fig. 3D).

**Fig. 2:**
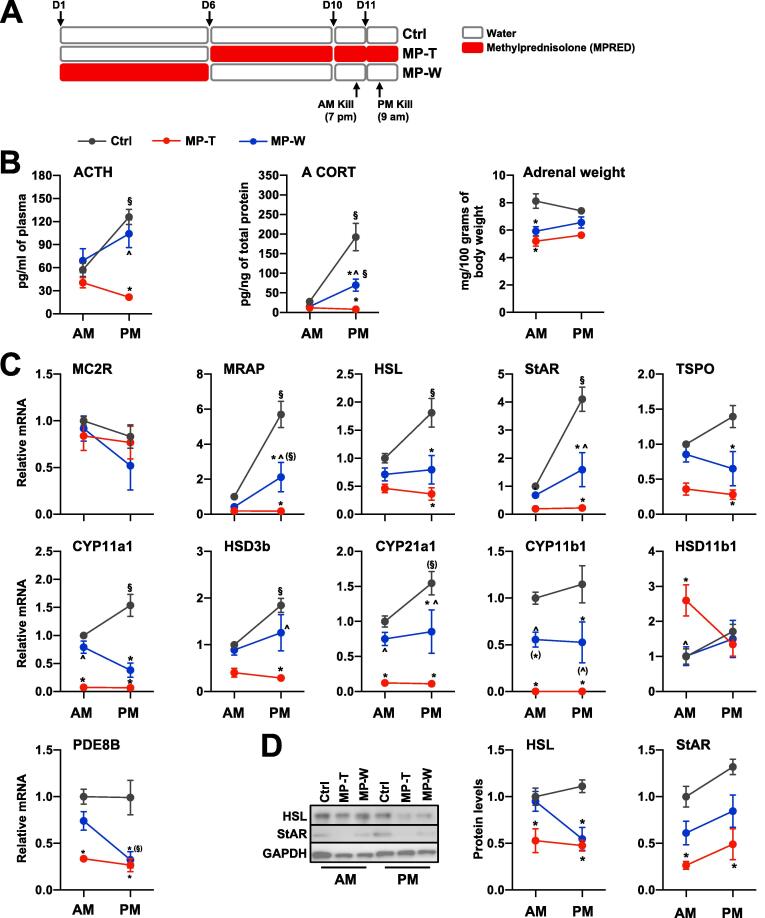
Effect of MPRED treatment and recovery on HPA axis and adrenal steroidogenic pathway. (A) Schematic representation of the AM and PM experiments. Plasma ACTH, adrenal corticosterone levels and relative adrenal weight (normalised to 100 g of body weight) (B), relative expression of steroidogenic genes mRNA (C) and steroidogenic proteins (D) were measured at 09:00 (AM) and at 19:00 (PM) in untreated rats (control group, Ctrl), rats treated with methylprednisolone (MPRED) in the drinking water (1 g/L) for 5 days (MPRED treatment group, MP-T), and rats treated with MPRED for 5 days and then left to recover for 5 days (MPRED withdraw group, MP-W). All data are mean ± SEM of 5–8 rats/group and are expressed as fold induction of AM-Ctrl. Data were analysed by two-way ANOVA and Tukey post-hoc test. *P < 0.05 *vs* Ctrl at the same time of day (effect of treatment); ^P < 0.05 *vs* MPT at the same time of day (effect of treatment); ^§^P < 0.05 *vs* AM of the same treatment group (effect of time of day). Symbols in parentheses indicate a tendency to significance (P < 0.10).

Interestingly, although we found no changes in pituitary POMC mRNA, the effects of MPRED on corticosterone secretion were associated with reduced basal ACTH secretion in the PM (*time × treatment*: F_(2, 32)_ = 7.042; P = 0.003) as well as with reduced adrenal corticosterone levels (*time × treatment*: F_(2, 32)_ = 17.43; P < 0.00001) and adrenal weight (*treatment*: F_(2, 32)_ = 18.45; P < 0.00001) in the PM and in the AM, respectively (Fig. 2B). However, while ACTH levels returned to normal after 5 days recovery from MPRED treatment, both adrenal corticosterone levels and adrenal weight were reduced in MP-W rats. Detailed results of statistical analysis are reported in Suppl. Table 3.

### 3.2 Reduced corticosterone secretion in rats treated with MPRED is associated with decreased expression of adrenal steroidogenic proteins

The observation that corticosterone levels are reduced both during the MPRED treatment and after 5 days withdraw lead us to investigate the effect of MPRED treatment on the adrenal steroidogenic pathway (Fig. 2C and D). We found that MPRED treatment affected the relative expression of MRAP mRNA (*time × treatment*: F_(2, 32)_ = 16.05; P < 0.00001), HSL mRNA (*time × treatment*: F_(2, 32)_ = 4.79; P = 0.0173), StAR mRNA (*time × treatment*: F_(2, 32)_ = 15.68; P=<0.00001), TSPO mRNA (*treatment*: F_(2, 32)_ = 23.1; P < 0.00001), Cyp11a1 mRNA (*time × treatment*: F_(2, 32)_ = 10.46; P < 0.00001), HSD3b1 mRNA (*time × treatment*: F_(2, 32)_ = 4.02; P = 0.03), Cyp21a1 mRNA (*treatment*: F_(2, 32)_ = 32.04; P < 0.00001) and Cyp11b1 mRNA (*treatment*: F_(2, 32)_ = 39.87; P < 0.00001), with a reduction in both MP-T and MP-W rats (Fig. 2C). Interestingly, an increase in HSD11b1 mRNA in the in MP-T rats was also observed (*time × treatment*: F_(2, 32)_ = 4.91; P = 0.015), suggesting that reactivation of corticosterone from its metabolite 11-dehydrocorticosterone may be a compensatory mechanism to overcome the lack of steroidogenic activity, due to reduced ACTH and subsequent decreased steroidogenic genes expression. This effect was found to be specific to the adrenal gland, as HSD11b1 expression in the liver of MP-T rats was unaffected (Suppl. Fig. 4). In addition, a reduction in the expression of the gene encoding for PDE8b the main phosphodiesterase expressed in the adrenal regulating the levels of cAMP (Tsai et al., 2011), was also observed in MP-T and MP-W rats (*time × treatment*: F_(2, 32)_ = 24.44; P < 0.00001). These findings suggest that reduced basal and ACTH-induced adrenal activity, and thus corticosterone secretion, in MP-W rats is due to decreased activity of the whole steroidogenic pathway. The effects of MPRED on steroidogenic gene expression in MP-T and MP-W rats also lead to significant changes in their AM/PM expression pattern. Specifically, MPRED treatment resulted in a loss of AM/PM variation in MRAP, HSL, StAR and HSD3b1 mRNA. The effects of MPRED on HSL and StAR mRNA were also found in the expression of their respective proteins (Fig. 2D), with HSL and StAR proteins decreased in MP-T rats, and not fully recovered in MP-W rats (HSL *treatment*: F_(2, 32)_ = 9.51; P = 0.001; StAR *treatment*: F_(2, 32)_ = 13.77; P < 0.00001; StAR *time*: F_(1, 32)_ = 7.36; P = 0.011). Detailed results of statistical analysis are reported in Suppl. Table 3.

### 3.3 MPRED affects the expression of genes encoding for adrenal transcription factors and regulators of steroidogenic gene expression and function

The transcription of adrenal steroidogenic genes is regulated by a number of transcription factors, nuclear receptors and regulators, and the activity and expression of most of these are also under the control of ACTH. Specifically, ACTH-induced phosphorylation of CREB require the activation of several nuclear receptors and co-regulators, including members of the CRTC family (CRTC1-3) (Smith et al., 2019), Nur77 (Martin et al., 2008) and steroidogenic factor 1 (SF-1) (Sugawara et al., 1996), and the repression of a number of negative regulators, including salt inducible kinase 1 (SIK1) (Takemori and Okamoto, 2008) and dosage sensitive sex-reversal, adrenal hypoplasia (Zazopoulos et al., 1997). Interestingly, GCs can inhibit the transcription of steroidogenic genes through a mechanism that involves increase in the expression of the steroidogenic co-repressor dosage-sensitive sex reversal, adrenal hypoplasia critical region, on chromosome X, gene 1 (DAX-1) (Gummow et al., 2006) as well as by inhibiting both the transcription and the activity of Nur77 via a mechanism that involves active GR (Song et al., 2004, Martin and Tremblay, 2008).

**Fig. 3:**
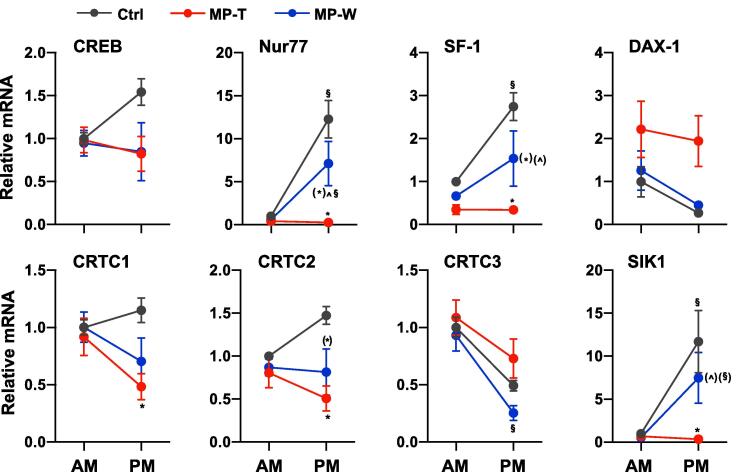
Effect of MPRED treatment and recovery on adrenal steroidogenic genes transcriptional regulators. The relative expression of mRNA of genes encoding for transcription factors regulating the transcription of genes within the steroidogenic pathway was measured at 9 AM and at 5 PM in the adrenal gland of untreated rats (control group, Ctrl), rats treated with methylprednisolone (MPRED) in the drinking water (1 g/L) for 5 days (MPRED treatment group, MP-T), and rats treated with MPRED for 5 days and then left to recover for 5 days (MPRED withdraw group, MP-W). Data are mean ± SEM of 5–8 rats/group and are expressed as fold induction of AM-Ctrl. Data were analysed by two-way ANOVA and Tukey post-hoc test. *P < 0.05 *vs* Ctrl at the same time of day (effect of treatment); ^P < 0.05 *vs* MP-T at the same time of day (effect of treatment); ^§^P < 0.05 *vs* AM of the same treatment group (effect of time of day). Symbols in parentheses indicate a tendency to significance (P < 0.10).

Therefore, to investigate the mechanism underlying the changes in steroidogenic genes and proteins induced by MPRED, we measured the expression of genes encoding for transcription factors that are known to be involved in the regulation of steroidogenic genes expression as described above. Consistent with the effects of MPRED treatment on steroidogenic genes expression, we found changes in Nur77 mRNA (*time × treatment*: F_(2, 32)_ = 10.35; P < 0.00001), SF-1 mRNA (*time × treatment*: F_(2, 32)_ = 4.89; P = 0.015), DAX-1 mRNA (*time × treatment*: F_(2, 32)_ = 6.28; P = 0.006), CRTC1 mRNA (*treatment*: F_(2, 32)_ = 4.03; P = 0.029), CRTC2 mRNA (*time × treatment*: F_(2, 32)_ = 3.68; P = 0.039), CRTC3 mRNA (*time × treatment*: F_(2, 32)_ = 24.44; P < 0.00001) and SIK1 mRNA (*time × treatment*: F_(2, 32)_ = 5.11; P = 0.013) (Fig. 3). To our surprise, none of the genes encoding for steroidogenic transcription factors or transcriptional co-regulators were either up- or down-regulated after withdrawing the MPRED treatment. As observed for steroidogenic genes expression, we found significant changes in the AM/PM expression of genes encoding for adrenal transcription factors. A loss in AM/PM difference was observed in Nur77, SF-1 and SIK1 mRNA in MP-T rats, whereas a gain in AM/PM difference was observed in CRTC3 mRNA in MP-W rats (AM > PM). Detailed results of statistical analysis are reported in Suppl. Table 3.

### 3.4 MPRED affects the expression of adrenal clock genes

**Fig. 4:**
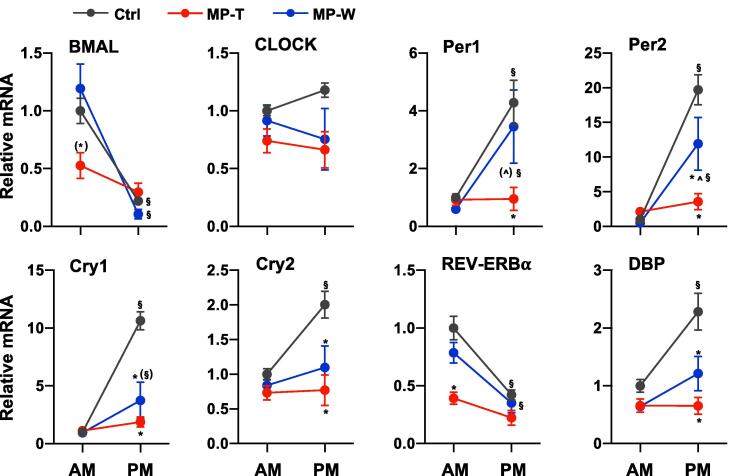
Effect of MPRED treatment and recovery on adrenal clock genes. The relative expression of mRNA of clock genes was measured in the adrenal gland of untreated rats (control group, Ctrl), rats treated with methylprednisolone (MPRED) in the drinking water (1 g/L) for 5 days (MPRED treatment group, MP-T), and rats treated with MPRED for 5 days and then left to recover for 5 days (MPRED withdraw group, MP-W) collected at 9 AM and at 5 PM. Data are mean ± SEM of 5–8 rats/group and are expressed as fold induction of AM-Ctrl. Data were analysed by two-way ANOVA and Tukey post-hoc test. *P < 0.05 *vs* Ctrl at the same time of day (effect of treatment); ^P < 0.05 *vs* MP-T at the same time of day (effect of treatment); ^§^P < 0.05 *vs* AM of the same treatment group (effect of time of day). Symbols in parentheses indicate a tendency to significance (P < 0.10).

As observed in the brain and in other peripheral organs, the adrenal gland expresses clock genes, and, as shown in this study and consistent with our previous report (Park et al., 2013), the expression of many steroidogenic genes and their transcriptional regulators is characterised by AM/PM differences. The crosstalk between glucocorticoids, clock genes and steroidogenic genes is complex. In vivo studies have shown that treatment with SGCs alters the pattern of circadian clock gene expression in various peripheral tissues (Balsalobre et al., 2000, Sujino et al., 2012). The molecular mechanisms underlying the effect of GCs on clock gene expression have been addressed by several groups and there is evidence that GCs regulate the expression of clock genes, including enhanced expression of Period 1 (Per1) and reduced expression of REV-ERBα (Torra et al., 2000), via the glucocorticoid receptor (GR) pathway (Nader et al., 2010). In turn, GR activity can be regulated by clock proteins, including CLOCK and Cryptochrome 1 and 2 (Cry1 and Cry2) (Nader et al., 2009, Lamia et al., 2011) via non-genomic mechanisms. With regard to a link between clock genes and adrenal steroidogenic pathways, studies in mice have shown that the expression of StAR is regulated by the activity of the CLOCK-BMAL complex (Son et al., 2008, Leliavski et al., 2014) and mutation in the *Bmal* gene in the adrenal gland leads to loss of corticosterone circadian rhythm in mice housed in altered light cycle (Son et al., 2008, Engeland et al., 2018). In light of this, we hypothesised that the changes in the expression of steroidogenic genes observed in MP-T and in MP-W rats may be associated with changes in adrenal clock genes expression. We found that MPRED treatment affected the expression of all clock genes studied (Fig. 4). Specifically, we found a decrease in the expression of BMAL mRNA (*time × treatment*: F_(2, 32)_ = 5.85; P < 0.008), CLOCK mRNA (*treatment*: F_(2, 32)_ = 4.10; P = 0.028), Per1 mRNA (*time × treatment*: F_(2, 32)_ = 4.53; P = 0.02), Per2 mRNA (*time × treatment*: F_(2, 32)_ = 12.87; P < 0.00001), Cry1 mRNA (*time × treatment*: F_(2, 32)_ = 24.04; P < 0.00001), Cry2 mRNA (*time × treatment*: F_(2, 32)_ = 4.41; P = 0.022), REV-ERBα mRNA (*treatment*: F_(2, 32)_ = 11.94; P < 0.00001) and DBP mRNA (*time × treatment*: F_(2, 32)_ = 5.93; P = 0.007) mRNA. Furthermore, a decrease in Per2, Cry1, Cry2 and DBP mRNA expression was found in MP-W rats. The effect of MPRED treatment on the expression of clock genes in the adrenal also led to changes in their AM/PM pattern. AM/PM variation was lost in BMAL Per1, Per2, Cry1, Cry2, REV-ERBα and DBP mRNA of MP-T rats, and in Cry1, Cry2 and DBP mRNA of MP-W rats. It is noteworthy that MPRED treatment affected clock gene expression in the liver differently (Suppl. Fig. 5) with no effects of treatment on Cry1, Cry2, REV-ERBbα and DBP. Detailed results of statistical analysis are reported in Suppl. Table 3.

### 3.5 MPRED reduces glucocorticoid response to inflammation

**Fig. 5:**
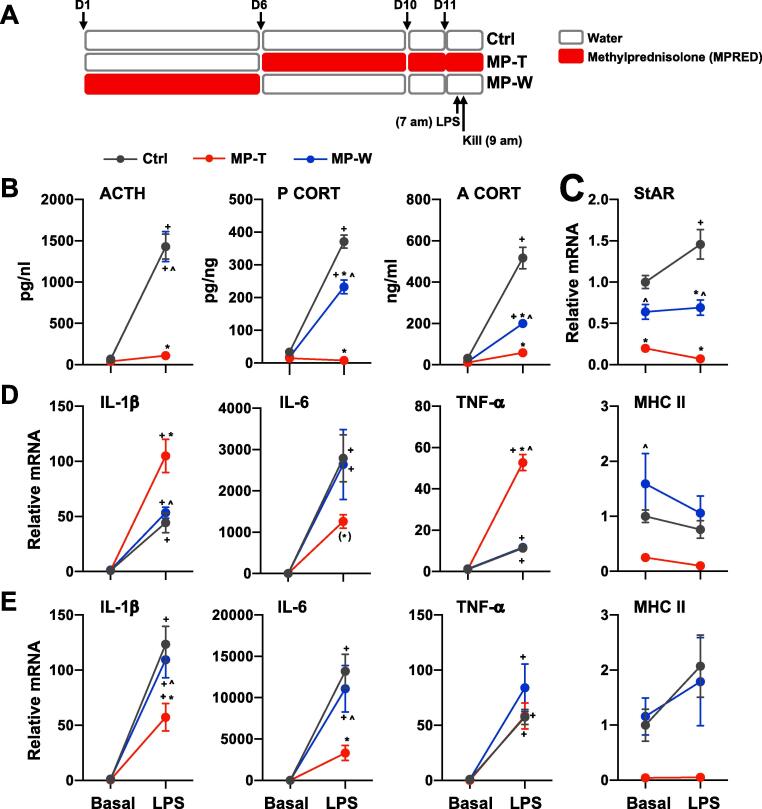
MPRED treatment reduces corticosterone response to LPS and increases adrenal cytokines mRNA levels. (A) Schematic representation of the LPS injection experiments. (B) Plasma ACTH and corticosterone (P CORT), and adrenal corticosterone (A CORT), (C) adrenal StAR mRNA (D) adrenal and (E) liver pro-inflammatory cytokines IL-1β, IL-6 and TNFα mRNA were measured 2 h after LPS injection in untreated rats (control group, Ctrl), rats treated with methylprednisolone (MPRED) in the drinking water (1 g/L) for 5 days (MPRED treatment group, MP-T), and rats treated with MPRED for 5 days and then left to recover for 5 days (MPRED withdraw group, MP-W). Data are mean ± SEM of 5–6 rats/group and are expressed as fold induction of Basal-Ctrl. Data were analysed by two-way ANOVA and Tukey post-hoc test. ^+^P < 0.05 *vs* LPS-untreated of the same MP treatment group (effect of LPS); *P < 0.05 *vs* Ctrl + LPS (effect of treatment); ^^^P < 0.05 *vs* MP + LPS (effect of treatment). Symbols in parentheses indicate a tendency to significance (P < 0.10).

One of the characteristics of SGCs-induced adrenal insufficiency is the inability of the adrenal gland to mount an appropriate response to stress and inflammation. In our previous experiment we showed that the corticosterone response to ACTH is reduced in both MP-T and MP-W rats (Fig. 1C). To estimate the functional relevance of decreased adrenal activity following MPRED treatment we next tested whether MP-T and MP-W rats also have a reduced adrenal response to acute inflammatory stress. Following MPRED treatment rats were injected with LPS as shown in Fig. 5A. We found that LPS-induced ACTH increase was reduced in MP-T rats, whereas ACTH levels were not different between Ctrl and MP-W rats (*LPS × treatment*: F_(2, 34)_ = 31.42; P < 0.00001). However, both plasma corticosterone (*LPS × treatment*: F_(2, 34)_ = 112.27; P < 0.00001) and adrenal corticosterone (*LPS × treatment*: F_(2, 34)_ = 47.26; P < 0.00001) levels were significantly reduced in MP-T and in MP-W rats in response to LPS (Fig. 5B), suggesting that the reduced corticosterone response to LPS depends on MPRED-induced changes in the adrenal gland. Indeed, StAR mRNA expression in response to LPS was also reduced in both MP-T and MP-W rats (*LPS × treatment*: F_(2, 34)_ = 4.70; P = 0.017; Fig. 5C). Detailed results of statistical analysis are reported in Suppl. Table 3.

### 3.6 MPRED increases intra-adrenal pro-inflammatory cytokines expression

Because LPS can affect the adrenal gland both directly as well as by inducing pro-inflammatory cytokines secretion from circulating immune cells (Kanczkowski et al., 2015), we measured the expression of intra-adrenal pro-inflammatory cytokines mRNA (Fig. 5B). We found that MPRED was able to sensitize the adrenal to release pro-inflammatory cytokines in response to LPS, as shown by higher IL-1β mRNA (*LPS × treatment*: F_(2, 32)_ = 8.84; P = 0.001) and TNFα mRNA (*LPS × treatment*: F_(2, 34)_ = 67.67; P < 0.00001) mRNA levels in MP-T rats, compared to Ctrl and MP-W rats (Fig. 5D and Suppl. Table 2). In contrast, no significant increase in IL-6 mRNA was observed in MP-T rats, while a significant increase was observed in both Ctrl and MP-W rats (*LPS*: F_(2, 34)_ = 212.09; P < 0.00001). This suggests that MPRED, in parallel to reducing hormonal response to LPS - presumably by decreasing the systemic inflammatory response to LPS - can also exert pro-inflammatory effects within the adrenal, as shown by an increased level of intra-adrenal pro-inflammatory cytokines. To assess whether the increased cytokine response in MP-T rats was associated with increased microphages in the adrenal following LPS injection, we measured the expression of the macrophage marker MHC II. We found that, while MPRED treatment decreased MHC II mRNA expression in MP-T rats (*treatment*: F_(1, 34)_ = 8.52; P = 0.001), there was no effect of LPS injection on MHC II mRNA across the experimental groups (Fig. 5D), suggesting that the pro-inflammatory effect of MPRED is exerted at the level of the adrenal and not within circulating macrophages. Pro-inflammatory effects of glucocorticoids have been previously shown in other cell type and tissue, including immune cells, brain areas, pituitary and liver (Frank et al., 2015). Therefore, we investigated whether the pro-inflammatory effects of MPRED were specifically occurring in the adrenal gland or similar effects could be seen in the liver. We found that, in contrast with what was observed in the adrenal gland, MPRED treatment reduced IL-1β mRNA and IL-6 mRNA response to LPS in the liver (IL-1β: *LPS × treatment*: F_(2, 30)_ = 4.67; P = 0.019; IL-6: *LPS × treatment*: F_(2, 30)_ = 5.45; P = 0.011), while no difference in TNFα mRNA was observed between the three groups (Fig. 5E). Detailed results of statistical analysis are reported in Suppl. Table 3.

### 3.7 MPRED increases intra-adrenal expression of pro-inflammatory factors

**Fig. 6:**
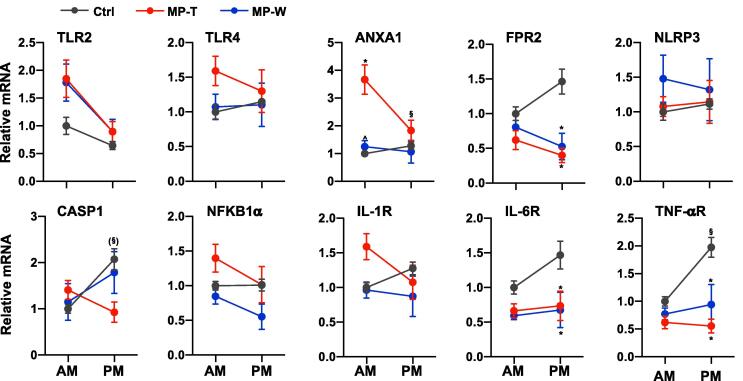
Effect of MPRED treatment and recovery on adrenal inflammatory factors. The relative expression of genes encoding for key inflammatory markers and modulators was measured at 9 AM and at 5 PM in the adrenal gland of untreated rats (control group, Ctrl), rats treated with methylprednisolone (MPRED) in the drinking water (1 g/L) for 5 days (MPRED treatment group, MP-T), and rats treated with MPRED for 5 days and then left to recover for 5 days (MPRED withdraw group, MP-W). Data are mean ± SEM of 5–8 rats/group and are expressed as fold induction of AM-Ctrl. Data were analysed by two-way ANOVA and Tukey post-hoc test. *P < 0.05 *vs* Ctrl at the same time of day (effect of treatment); ^§^P < 0.05 *vs* AM of the same treatment group (effect of time of day). Symbols in parentheses indicate a tendency to significance (P < 0.10).

To investigate the mechanisms underlying the observed pro-inflammatory effects of MPRED in the adrenal – that is increased intra-adrenal IL-1β and TNFα mRNA in response to LPS – we measured the basal expression of genes encoding for modulators of the immune response (Fig. 6). Overall, we found an effect of MPRED on TLR2 mRNA (*treatment*: F_(2, 32)_ = 3.43; P = 0.047), ANXA1 mRNA (*time × treatment*: F_(2, 32)_ = 6.20; P = 0.006), FPR2 mRNA (*time × treatment*: F_(2, 32)_ = 3.84; P = 0.034), Casp1 mRNA (*time × treatment*: F_(2, 32)_ = 3.86; P = 0.034) and NFKB1α mRNA (*time × treatment*: F_(2, 32)_ = 5.12; P = 0.013), as well as on IL-6R mRNA (*treatment*: F_(2, 32)_ = 8.83; P = 0.001), and TNFαR mRNA (*time × treatment*: F_(2, 32)_ = 4.98; P = 0.014). Post-hoc test revealed a significant increase in ANXA1 mRNA in the AM in MP-T rats, and a significant decrease in FPR2 mRNA in the PM of both MP-T and MP-W rats. These data suggest that the pro-inflammatory effects of MPRED in the adrenal may be mediated by an increase in TLR expression, as well as an increase in the inflammatory mediator Annexin 1 (encoded by the *Anxa1* gene). This hypothesis is further supported by the observation that the anti-inflammatory effects of MPRED in the liver of MP-T rats were associated with changes in TLR2 mRNA (*treatment*: F_(2, 31)_ = 17.60; P < 0.00001), TLR4 mRNA (*treatment*: F_(2, 31)_ = 9.13; P = 0.001), NLRP3 mRNA (*treatment*: F_(2, 31)_ = 18.08; P < 0.00001), Casp1 mRNA (*treatment*: F_(2, 31)_ = 8.60; P = 0.001), and NFKB1α mRNA (*treatment*: F_(2, 31)_ = 10.26; P = 0.001), with a decreased expression of TLR2 and NLPR3 mRNA in MP-T rats, and no effects on MP-W rats (Suppl. Fig. 5). MPRED also induced changes in liver IL-1R mRNA (*treatment*: F_(2, 31)_ = 8.30; P = 0.002), IL-6R mRNA (*time × treatment*: F_(2, 32)_ = 4.53; P = 0.021), and TNFαR (*treatment*: F_(2, 31)_ = 3.45; P = 0.047) (Suppl. Fig. 5). Detailed results of statistical analysis are reported in Suppl. Table 3.

## 4 Discussion

In this study we have provided a detailed analysis of the effects of the synthetic glucocorticoid MPRED on HPA axis activity and, importantly, on genes that directedly or indirectly regulate steroid biosynthesis in the adrenal gland. Our data suggest a key role of proteins regulating ACTH signalling (MRAP), cholesterol levels (HSL) and intra-mitochondria availability (StAR and TSPO), as well as cholesterol enzymatic processing to corticosterone (CYP11a1, CYP21 and CYP11b1) in the disruption of adrenal responsiveness observed in conditions of MPRED-induced adrenal insufficiency. Furthermore, we provide evidence for a decreased adrenal responsiveness to both ACTH and LPS stimulation after 5 days MPRED treatment as well as after 5 days withdrawal from MPRED. Importantly, we show that the downregulation of the adrenal gland observed after 5 days treatment are associated with a pro-inflammatory effect of MPRED that appears to be specific to the adrenal gland.

Clinical therapy with high doses of SGC results in adrenal insufficiency, characterised by adrenal atrophy and decreased basal and stress-induced cortisol secretion, that may persist for over 3 years after SGC withdrawal (Joseph et al., 2016). Here we show that rats treated with MPRED for 5 days show decreased adrenal weight and cortisol secretion, both in basal and stress conditions, even 5 days after withdrawing from the MPRED treatment. Interestingly, in addition to reduced hormone levels in recovering rats (MP-W group), pulsatile corticosterone pattern is also affected with decreased pulse length, inter-pulse interval and pulse frequency. The effects of MPRED treatment on pulse characteristics observed in the MP-W groups are not surprising. Pulsatile corticosterone secretion depends both on dynamic responses of the adrenal steroidogenic pathway to pulses of ACTH (Spiga et al., 2017), as well as dynamic interactions between GC negative feedback within the pituitary corticotroph cells and the hypothalamic secretion of CRH (Walker et al., 2010, Walker et al., 2012). Therefore, it is plausible to speculate that long-term exposure to SGC may have affected GC signalling in the anterior pituitary, and thus affected the feedforward-feedback mechanisms regulating corticosterone pulsatility. Based on our mathematical modelling and *in vivo* work, changes in GC-regulated negative feedback within pituitary corticotroph cells will impact the dynamics of ACTH and, thus, GC secretion (Walker et al., 2010, Walker et al., 2012). Although AM and PM ACTH levels were unaffected following MPRED withdraw, it is possible that changes in the dynamics of ACTH (e.g. length and frequency) may have occurred, resulting in the observed changes in corticosterone pulsatility. We have shown in a previous study that intravenous infusion of pulsatile ACTH in rats with MPRED-suppressed HPA axis was able to recover steroidogenic function in the adrenal (Spiga et al., 2011). Here we found that despite apparently normal ACTH levels, steroidogenic function is still downregulated in MP-W rats, further indicating that ACTH secretion in these rats is not completely restored (i.e. changes in ACTH dynamics), and a full recovery of adrenal function (and ACTH dynamic) may only occur after a longer withdrawal period.

In addition to this, we have recently shown that dynamics within the adrenal steroidogenic pathway are crucial for normal ultradian glucocorticoid secretion, and that disruption of these dynamics will result in disrupted glucocorticoid synthesis (Spiga et al., 2017). Although in this study adrenal steroidogenic gene expression was only measured at two times of day it did reveal changes in their circadian pattern of expression, supporting the speculation that that the observed circadian changes are associated with ultradian changes which, in turn, affect the dynamics of hormone synthesis and secretion. This is compatible with our model of the pulse generator, in which pulsatility within the pituitary-adrenal system not only depends on the feedback inhibition, but also on the feedforward drive mediated by ACTH. Here the delay in the adrenal response to ACTH play a crucial role in out model, and such delay can be affected by changes within the steroidogenic pathway that we have observed here in both MP-T and MP-W rats. It is noteworthy that, while changes in GR and MR gene expression were not observed in the pituitary, increased GR expression was found in the hypothalamus of MP-W rats. The observed effect of MPRED on hypothalamic GR could be due to decreased occupancy of GR since the closely related prednisolone is a substrate of the multidrug resistance (mdr) P-glycoprotein (Pgp) (Karssen et al., 2002). If MPRED, at least at the doses used in this study, did not cross the blood brain barrier, it would not be able to access the brain itself- as also suggested by a lack of effect of MPRED on the GR-responsive genes SGK1 and GILZ in the hypothalamus (data not shown).

Interestingly, adrenal corticosterone levels were also reduced in both groups, suggesting that 1) reduced plasma corticosterone levels are the result of reduced corticosterone synthesis in the adrenal gland, and 2) although a reduction in circulating levels of ACTH may be responsible for reduced basal corticosterone secretion during the MPRED treatment in MP-T rats, a direct effect of MPRED within the adrenal steroidogenic pathway may be driving the long-term inhibition of corticosterone secretion observed after MPRED withdraw in MP-W rats. This is further supported by our findings that adrenal responsiveness to exogenous ACTH is still reduced in MP-W rats. Tissue availability of glucocorticoids depends on their binding to the transporter protein corticosteroid binding globulin (CBG). Consistent with previous evidence (Smith and Hammond, 1992), liver CBG mRNA expression was highly reduced in MP-T rats whereas it was unaffected in MP-W rats (Suppl. Fig. 7), suggesting that the decreased levels of plasma corticosterone in this group will result in reduced hormone levels at tissue level.

The increase in GCs secretion upon ACTH stimulation is rapid (<5 min); however, because of their lipophilic nature, GCs cannot be pre-stored in vesicles but must be *de novo* synthesized from its precursor, cholesterol. This process requires ACTH-mediated non-genomic mechanisms involving the rapid activation of steroidogenic proteins, including hormone-sensitive lipase (HSL) and steroidogenic acute regulatory (StAR) proteins, which regulate cellular availability and intra-mitochondrial levels or cholesterol, respectively (Lin et al., 1995, Kraemer and Shen, 2002). ACTH also activates mitochondrial steroidogenic enzymes belonging to the cytochrome P450 (CYP) and hydroxysteroid dehydrogenase (HSD) families, which, in the rat, convert cholesterol into corticosterone (Midzak and Papadopoulos, 2016). In parallel, in order to maintain optimal steroidogenic response to further stimuli, ACTH simultaneously triggers genomic responses involving the transcription of genes encoding for the above-mentioned steroidogenic proteins and enzymes. We have previously shown that 5-days treatment with MP induces a reduction in the expression of steroidogenic genes in the adrenal gland (Spiga et al., 2011). In the present study we confirmed and expanded upon our previous work by showing that prolonged treatment with MPRED lead to changes in the expression of several genes encoding for steroidogenic proteins in the adrenal gland. A recent study has revealed that some steroidogenic gene promoter, including *Cyp11a1*, *Cyp11b1* and *HSDb2*, contains a negative GRE (Wang et al., 2019), and this suggests that their expression is directly regulated by MPRED (and the endogenous corticosterone). Other steroidogenic genes, including StAR and MC2R, are downregulated by GCs *via* increased expression of the negative regulator DAX-1 (Gummow et al., 2006). Notably, the effects of MPRED on StAR, TSPO and HSL mRNA appear to be specific to the adrenal gland, as opposite effects on these genes were observed in the liver (Suppl. Fig. 8).

The effects of MPRED on adrenal steroidogenic gene expression was paralleled by changes in the expression of their transcriptional regulators and in the expression of several key clock genes, suggesting that both direct and indirect effects of MPRED may be responsible for the changes in steroidogenic activity in the adrenal gland. It was noteworthy that MPRED treatment resulted in decreased adrenal expression of the clock gene Per1, and other genes known to be induced by glucocorticoids via activation of GR including GILZ and FKBP5 (Suppl. Fig. 9A), whereas these genes were all induced in the liver. The mechanisms underlying the differential effect of GR activation between the adrenal and the liver is not clear, and further studies on the MPRED-induced binding of GR to Per1 promoter in the adrenal would be needed to elucidate this.

While from our data it is clear that suppressed steroidogenic function after 5-days MPRED treatment may be a direct effect of the SGC treatment, the mechanisms underlying the effects observed after 5 days withdrawal are less clear. It is unlikely that circulating MPRED is still present and/or is still exerting direct effects due to relative short half-life of the hormone (approx. 2.5 h in male rats) (Ayyar et al., 2019b), as also suggested by rapid body weight gain upon removal of the treatment (Suppl. Fig. 1) and lack of effect on GC-responsive genes GR, FKBP5 and GILZ mRNA both in the adrenal and in the liver of MP-W rats (Suppl. Fig. 9A and B). The mechanisms through which MRED affect the steroidogenic pathway in the withdrawal period are not clear from our data, and further studies are needed to focus on GR activity and function within the adrenal gland.

One of the pathological consequences of SGC-induced adrenal insufficiency is the potential development of an adrenal crisis resulting from decreased endogenous GC secretion in response to inflammatory stressors such as infections, injuries and major surgery. Consistent with clinical data, administration of LPS in MP-T rats resulted in suppressed ACTH and corticosterone responses, due to the suppressive effect of MPRED. However, despite a normal ACTH response, both plasma and adrenal corticosterone response was reduced in MP-W rats, suggesting that, as observed in human patients, the effect of prolonged SGC treatment can still affect adrenal activity even after discontinuation of the treatment. It was interesting to observe that the adrenal expression of the pro-inflammatory cytokines IL-1β and TNFα in response to LPS was elevated in MP-T rats compared to Ctrl, while IL-6 expression was decreased. We were surprised to observe a discrepancy between the effects on IL-1β and TNFα, and on IL-6, and further studies on the mechanisms behind these differences are needed. However, Frank and colleagues have shown a similar result with corticosterone treatment increasing IL-1β and TNFα mRNA in hippocampal microglia in response to LPS, with no effect on IL-6 mRNA (Frank et al., 2010). Indeed, a pro-inflammatory effect of GC has been previously reported in immune cells, in the hippocampus and in the liver (Frank et al., 2010, Busillo et al., 2011, Frank et al., 2013). It is noteworthy that, despite a decrease in corticosterone response, the expression of adrenal cytokines in response to LPS was not affected in MP-W rats. Indeed, an increase in cytokine expression was expected, due to the well characterised reduced corticosterone-mediated anti-inflammatory effect. Nevertheless, a pro-inflammatory effect of MPRED in the adrenal during the treatment can have functional consequences. Pro-inflammatory cytokines, including IL-1β, IL-6 and TNFα, play a key role in the immune-adrenocortical cross-talk as they can directly modulate adrenal function (Kanczkowski et al., 2015). Indeed, IL-1β, IL-6 and TNFα can stimulate adrenal steroidogenesis *in vivo* in the rat (Spiga et al., 2017). Given that MPRED treatment decreased LPS-induced corticosterone secretion in both MP-T and MP-W rats, it is plausible to speculate that any effect of cytokines on corticosterone secretion would have been masked by the effects of MPRED. Furthermore, intra-adrenal activation of the immune system can also affect adrenal functionality by damaging adrenal endothelial microvascular cells (Chen et al., 2019). In addition to this, both synthetic glucocorticoids and pro-inflammatory cytokines can decrease adrenal steroidogenic function by inducing apoptosis and reducing cell viability (Mikhaylova et al., 2007, Finco et al., 2018).

The mechanisms underlying the pro-inflammatory effects of GCs are still matter of investigation and several mechanisms have been proposed, including (1) an increase expression of TLR and/or cytokines receptors, (2) an increase in macrophagic levels and/or activity, and (3) an increase in the expression of the NLP3 inflammasome component NLP3 and Casp1 (Busillo et al., 2011, Frank et al., 2014). Consistent with this evidence, we found that MPRED-induced pro-inflammatory response to LPS was associated with increased basal levels of both TLR2 and TLR4 expression in MP-T rats. TLR4 is the primary receptor for Gram-negative bacterial LPS, while TLR2 functions as the primary receptor for recognition of Gram-positive bacterial cell wall components. There is evidence that the cellular responses to LPS requires both TLR4 and TLR2 (Good et al., 2012), therefore, based on our findings, it is plausible to speculate that the increase in TLR2 mRNA expression may lead to increased activity of TLR4, thus increased adrenal responsiveness to LPS. Interestingly, MPRED induced the expected anti-inflammatory effects in the liver and this was associated with a significant decrease in both TLR2 and TLR4 mRNA expression. In addition, we found an increase ANXA1 mRNA, a GC-induced gene encoding for the inflammatory mediator Anexin1, in the adrenal. Previous studies have shown that a decrease in ANXA1 expression is linked to an increase in TLR4-mediated responses, a decrease in pro-inflammatory cytokines response to LPS and increased anti-inflammatory effects of GCs (Davies et al., 2007). Thus, this is consistent with the observed decrease in corticosterone response to LPS, and with increased expression of pro-inflammatory cytokines in MP-T rats. Notably, the expression of ANXA1 receptor FPR2 was decreased both in MP-T and MP-W rats, suggesting compensatory mechanisms within the Anexin1 pathways.

Glucocorticoids induce the formation of the inflammasome, a multiprotein signalling-complex that includes the GC-responsive gene nod-like receptor 3 (NLRP3) (Leemans et al., 2011). Assembly and activation of the NLRP3 inflammasome leads to activation of caspase 1 (encoded by the casp1 gene) which induces the cleavage of pro-IL-1β to its mature form IL-1β (Martinon et al., 2009). In the present study, we found no significant changes on the expression of either NLRP3 or CASP1 in the adrenal therefore it is unclear from our data whether the pro-inflammatory effects of SGC in the adrenal gland involve the inflammasome complex. LPS binding to TLR4 activates the NF-kB pathway and, in turn, SGCs repress inflammation by inducing the expression of the NF-kB repressor NFKB1α (Munhoz et al., 2010). Paradoxically, NFKB1α is also induced by NF-kB, thus an increase in NFKB1α can be used as a marker of pro-inflammatory effect of NF-kB (Sun et al., 1993). We show here a significant overall effect of MPRED on NFKB1α that was associated with a pattern of increase in MP-T rats, thus suggesting its involvement in the pro-inflammatory effect of SGC.

Unfortunately, experimental constraints precluded an assessment of the temporal changes in hormone secretion and gene expression following LPS injections (experiment 3), and of gene expression following ACTH administration (experiment 1). Our recent work has shown that the adrenal steroidogenic pathway responds dynamically to both ACTH and LPS perturbations (Spiga et al., 2017) and therefore it would be important to asses MPRED effects on steroidogenic activity not only in term of absolute changes but also in term of pattern of response. One other limitation of our data is that we only performed this study using male rats. Sexual dimorphism in basal HPA axis activity, as well as in its response to stress and in systemic response to inflammation has been well characterised. Furthermore, glucocorticoid receptor signalling has also been shown to be different between male and female rats (Goel et al., 2014). In addition, MPRED metabolism has been shown to be different in male and female rats (Ayyar et al., 2019a), therefore, we cannot exclude that MPRED may affect adrenal corticosterone dynamics and adrenal steroidogenesis differently in male and female rats. Therefore, based on the present data, future studies should be designed to take into consideration sex differences in adrenal activity and their underlying molecular mechanisms in the context of models of adrenal insufficiency.

In summary, in this study we have provided a thorough analysis of the effects of the synthetic glucocorticoid methylprednisolone on HPA axis activity and, importantly, on genes that directly or indirectly regulate steroid biosynthesis in the adrenal gland. Our data provide valuable insights on the regulation of the adrenal steroidogenic pathway that are important starting points for future studies on adrenal gland physiology. Importantly, the results of this study further expand our knowledge of the mechanisms through which synthetic glucocorticoids induce adrenal insufficiency, by showing simultaneous effects within multiple pathways involved in steroidogenesis, including circadian clock gene and inflammation pathways. Furthermore, we show here for the first time a pro-inflammatory effect of synthetic glucocorticoids in the adrenal gland. A more detailed understanding of the effects of synthetic glucocorticoids on HPA axis dynamics and on adrenal steroidogenic activity in basal conditions and in response to immune stress, and the identification of mechanisms regulating these effects, will help the development of more rational therapeutic strategies.
