# Supplementary material for: Prolonged treatment with the synthetic glucocorticoid methylprednisolone affects adrenal steroidogenic function and response to inflammatory stress in the rat
Source: Brain Behav Immun. 2020 Jul;87:703–14. doi: 10.1016/j.bbi.2020.03.001 (PMC7327516; doi:10.1016/j.bbi.2020.03.001)
Supplement: Supplementary data 2 [file mmc2.pdf]

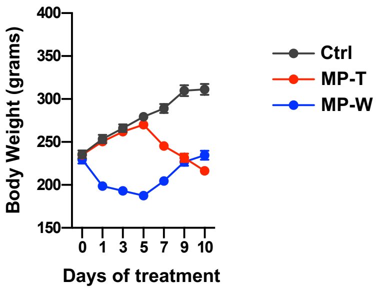

Supplementary Figure 1. Effect of MPRED treatment and recovery on body weight.

Body weight gain/loss during and after the treatment of untreated rats (control group, Ctrl), rats treated with methylprednisolone (MPRED) in the drinking water (1g/L) for 5 days (MPRED treatment group, MP-T), and rats treated with MPRED for 5 days and then left to recover for 5 days (MPRED withdraw group, MP-W). Rats were weighted at 9 am every two days. Data are mean ± SEM of 5-8 rats/group.

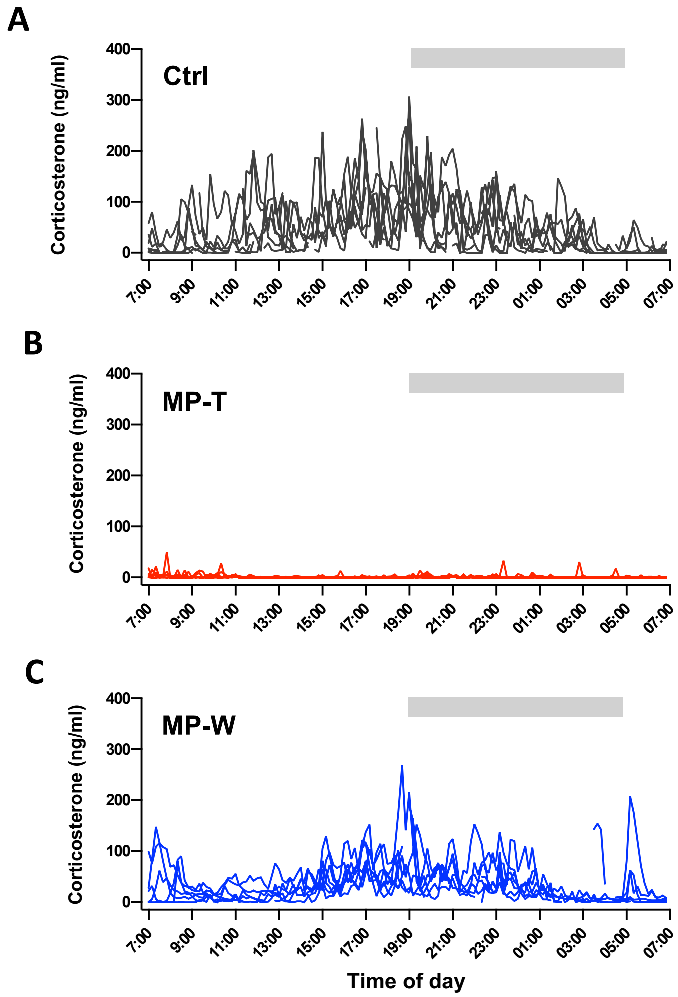

Supplementary Figure 2. Effect of MPRED treatment and recovery on basal and ACTH induced corticosterone secretion.

Individual corticosterone profiles from (**A**) untreated rats (control group, Ctrl; n=7), (**B**) rats treated with methylprednisolone (MPRED) in the drinking water (1g/L) for 5 days (MPRED treatment group, MP-T; n=6), and (**C**) rats treated with MPRED for 5 days and then left to recover for 5 days (MPRED withdraw group, MP-W; n=7). Blood samples were collected every 10 min from 07:00 of day 1 for 24 hours using an automated blood sampling system. Gray bar represents the dark period (19:00-05:00).

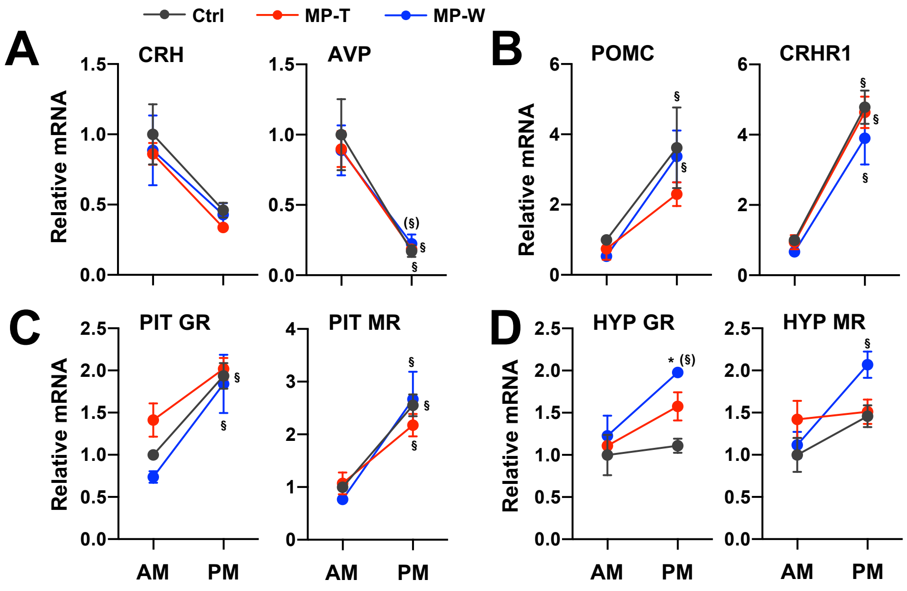

Supplementary Figure 3. Effect of MPRED treatment and recovery on HPA axis activity regulators.

The expression of hypothalamic CRH and AVP mRNA (A), anterior pituitary POMC, CRHR1, GR and MR mRNA (B and C), as well as hypothalamic GR and MR mRNA (D) was measured at 9 AM and at 5 PM in untreated rats (control group, Ctrl), rats treated with methylprednisolone (MPRED) in the drinking water (1g/L) for 5 days (MRED treatment group, MP-T), and rats treated with MPRED for 5 days and then left to recover for 5 days (MPRED withdraw group, MP-W). Data are mean ± SEM of 5-8 rats/group and are expressed as fold induction of AM-Ctrl. PIT, anterior pituitary; HYP, hypothalamus. Data were analysed by two-way ANOVA and Tukey post-hoc test. *P<0.05 *vs* Ctrl at the same time of day (effect of treatment); §P<0.05 *vs* AM of the same treatment group (effect of time of day). Symbols in parentheses indicate a tendency to significance (P < 0.10).

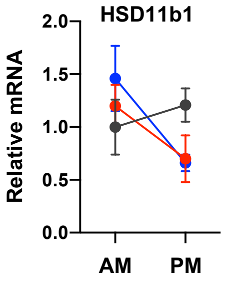

Supplementary Figure 4. Effect of MPRED treatment and recovery on the expression of HSD11b1 in the liver.

The expression of HSD11b1 mRNA was measured in the liver of untreated rats (control group, Ctrl), rats treated with methylprednisolone (MPRED) in the drinking water (1g/L) for 5 days (MPRED treatment group, MP-T), and rats treated with MPRED for 5 days and then left to recover for 5 days (MPRED withdraw group, MP-W) at 9 AM and at 5 PM. Data are mean ± SEM of 5-6 rats/group and are expressed as fold induction of AM-Ctrl.

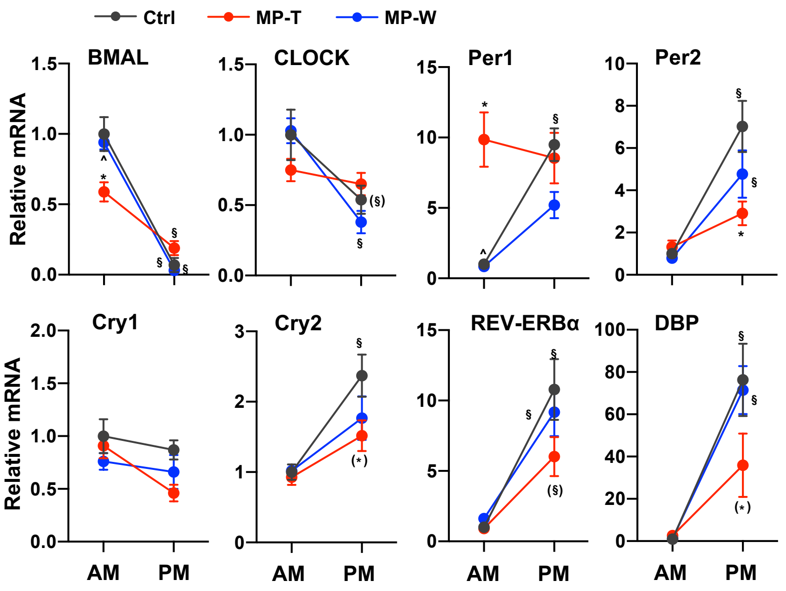

Supplementary Figure 5. Effect of MP treatment and recovery on liver clock genes.

The relative expression of mRNA of clock genes was measured in the liver of untreated rats (control group, Ctrl), rats treated with methylprednisolone (MPRED) in the drinking water (1g/L) for 5 days (MPRED treatment group, MP-T), and rats treated with MPRED for 5 days and then left to recover for 5 days (MPRED withdraw group, MP-W) collected at 9 AM and at 5 PM. Data are mean ± SEM of 5-6 rats/group and are expressed as fold induction of AM-Ctrl. Data were analysed by two-way ANOVA and Tukey post-hoc test. *P<0.05 *vs* Ctrl at the same time of day (effect of treatment); ^P<0.05 *vs* MP-T at the same time of day (effect of treatment); §P<0.05 *vs* AM of the same treatment group (effect of time of day). Symbols in parentheses indicate a tendency to significance (P < 0.10).

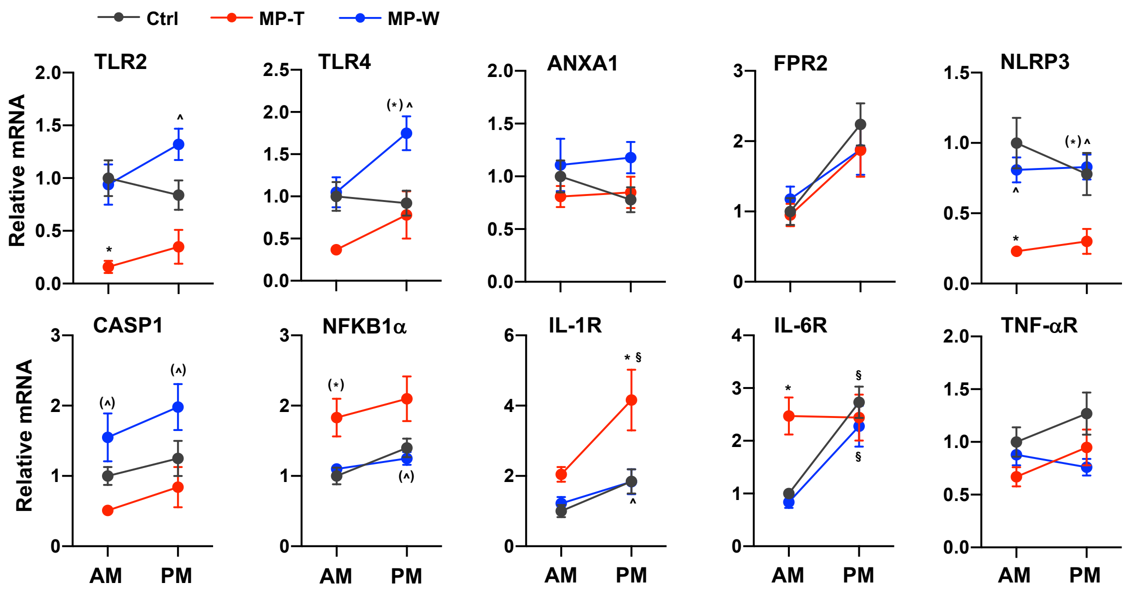

Supplementary Figure 6. Effect of MPRED treatment and recovery on liver inflammatory factors.

The relative expression of genes encoding for key inflammatory markers and modulators was measured at 9 AM and at 5 PM in the liver of untreated rats (control group, Ctrl), rats treated with methylprednisolone (MPRED) in the drinking water (1g/L) for 5 days (MPRED treatment group, MP-T), and rats treated with MPRED for 5 days and then left to recover for 5 days (MPRED withdraw group, MP-W) of Ctrl, MP-T and MP-W rats. Data are mean ± SEM of 5-6 rats/group and are expressed as fold induction of AM-Ctrl. Data were analysed by two-way ANOVA and Tukey post-hoc test. *$P<0.05$ *vs* Ctrl at the same time of day (effect of treatment); ^$P<0.05$ *vs* MP-T at the same time of day (effect of treatment); §$P<0.05$ *vs* AM of the same treatment group (effect of time of day). Symbols in parentheses indicate a tendency to significance ($P < 0.10$).

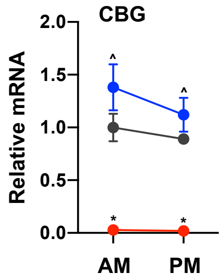

Supplementary Figure 7. Effect of MPRED treatment and recovery on the expression of corticosteroid binding globulin in the liver.

The expression of corticosteroid binding globulin (CBG) mRNA was measured in the liver of untreated rats (control group, Ctrl), rats treated with methylprednisolone (MPRED) in the drinking water (1g/L) for 5 days (MPRED treatment group, MP-T), and rats treated with MPRED for 5 days and then left to recover for 5 days (MPRED withdraw group, MP-W) at 9 AM and at 5 PM. Data are mean ± SEM of 5-6 rats/group and are expressed as fold induction of AM-Ctrl. Data were analysed by two-way ANOVA and Tukey post-hoc test. *$P<0.05$ *vs* Ctrl at the same time of day (effect of treatment). ^$P<0.05$ *vs* MP-T at the same time of day (effect of treatment).

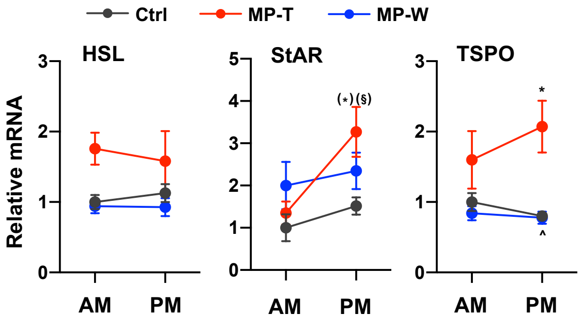

Supplementary Figure 8. Effect of MPRED treatment and recovery on liver steroidogenic genes.

The relative expression of genes encoding for steroidogenic genes was measured at 9 AM and at 5 PM in the liver of untreated rats (control group, Ctrl), rats treated with methylprednisolone (MPRED) in the drinking water (1g/L) for 5 days (MPRED treatment group, MP- T), and rats treated with MPRED for 5 days and then left to recover for 5 days (MPRED withdraw group, MP-W). Data are mean ± SEM of 5-6 rats/group and are expressed as fold induction of AM-Ctrl. Data were analysed by two-way ANOVA and Tukey post-hoc test. *P<0.05 *vs* Ctrl at the same time of day (effect of treatment); ^P<0.05 *vs* MPT at the same time of day (effect of treatment); §P<0.05 *vs* AM of the same treatment group (effect of time of day). Symbols in parentheses indicate a tendency to significance (P < 0.10).

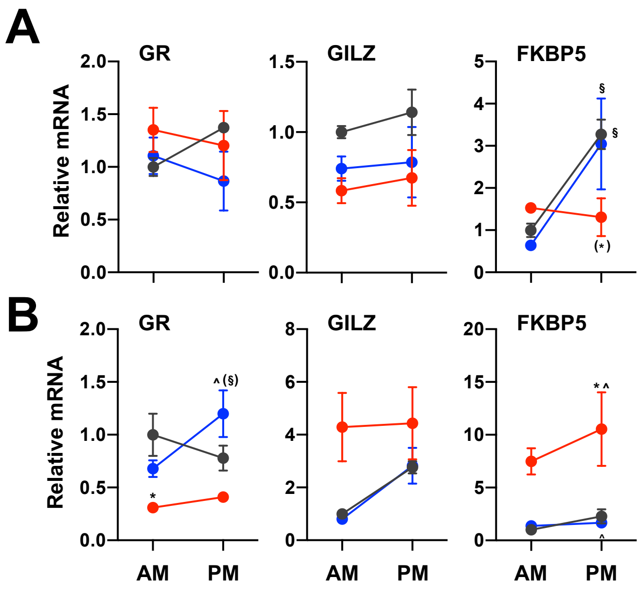

Supplementary Figure 9. Effect of MPRED treatment and recovery on GR-responsive genes.

The relative expression of genes encoding for GR and GR-responsive genes was measured at 9 AM and at 5 PM in the adrenal (**A**) and in the liver (**B)** of untreated rats (control group, Ctrl), rats treated with methylprednisolone (MPRED) in the drinking water (1g/L) for 5 days (MPRED treatment group, MP-T), and rats treated with MPRED for 5 days and then left to recover for 5 days (MPRED withdraw group, MP-W)**.** Data are mean ± SEM of 5-6 rats/group and are expressed as fold induction of AM-Ctrl. Data were analysed by two-way ANOVA and Tukey post-hoc test. *P<0.05 *vs* Ctrl at the same time of day (effect of treatment); ^P<0.05 *vs* MPT at the same time of day (effect of treatment); §P<0.05 *vs* AM of the same treatment group (effect of time of day). Symbols in parentheses indicate a tendency to significance (P < 0.10).
