# Supplementary material for: Prolonged treatment with the synthetic glucocorticoid methylprednisolone affects adrenal steroidogenic function and response to inflammatory stress in the rat
Source: Brain Behav Immun. 2020 Jul;87:703–14. doi: 10.1016/j.bbi.2020.03.001 (PMC7327516; doi:10.1016/j.bbi.2020.03.001)
Supplement: Supplementary data 3 [file mmc3.docx]

| **Supplementary Table 1: RTqPCR primers** | |  |
| --- | --- | --- |
| **Target** | **Forward primer** | **Reverse primer** |
| Actin | CACCCGCGAGTACAACCTTC | CCCATACCCACCATCACACC |
| ANXA1 | TCCCTGGAAGACAAGGCAATAC | GCCTCATCCACACCTTTAACCA |
| AVP | TGCCTGCTACTTCCAGAACTGC | AGGGGAGACACTGTCTCAGCTC |
| BMAL | TCCACAGCACAGGCTACTTGAA | TTGCAACGAGGCAGCTCAGAT |
| CASP1 | ATGCCGTGGAGAGAAACAAG | CCAGGACACATTATCTGGTG |
| CBG | AAGCTTATGACCCCAAGGCAG | GGTTCAGAGCGCAGGTGTAG |
| CLOCK | GACAGCCCCACTGTACAATACG | TGCGGCATACTGGATGGAAT |
| CRH | CTCTCTGGATCTCACCTTCCAC | CTAAATGCAGAATCGTTTTGGC |
| CRHR1 | CTTCTCCTTCTGGGGCTGA | AGGTGCCAATGAGGTCCAC |
| CRTC1 | CACCAGAGCACAATGACACC | GCCTTCTTTGAGTCCCATGA |
| CRTC2 | CCCACCCCAAAGTCTCTACA | CCCCAGGCTGAAGTCATTT |
| CRTC3 | AAGCCAGGTACCCTCCAACT | GCACATACAGGAAAGCAGCA |
| Cry1 | AAGTCATCGTGCGCATTTCA | TCATCATGGTCGTCGGACAGA |
| Cry2 | TGGATAAGCACT TGGAACGGAA | TGTACAAGTCCCACAGGCGGTA |
| CYP11a1 | TGCGAGGGTCCTAACCCGGA | ACCTTCCAGCAGGGGCACGA |
| CYP11b1 | CAGAGGGTCGCCAACAGTC | TGAACATCTGGGTTCCGAGC |
| CYP21a1 | CCGGGGTTTCTGCACTTTCT | CCACCACATCTTGTAGCCCC |
| DAX-1 | TCCAGGCCATCAAGAGTTTC | GTGCTCAGTGAGGATCTGC |
| DBP | CCTTTGAACCTGATCCGGCT | TGCCTTCTTCATGATTGGCTG |
| FKBP5 | GAACCCAATGCTGAGCTTATG | ATGTACTTGCCTCCCTTGAAG |
| FPR2 | TGAGGACTCCCAAGCGTACT | CCCAGTTCCGGTGTTGGTTT |
| GILZ | CCATGGATCTAGTGAAGAATCATTTG | CCACCTCCTCTCTCACAGCATAC |
| GR | CACAGCTCACCCCTACCTTG | TTTCTGAAGCCTGGTATCGCC |
| HSD3b1 | AACCAAGGAGGAATTCTCTAAGCTG | TGGCACACTGGCTTCGAC |
| HSD11b1 | TCTGTGAGCCCTACCCACAAA | CATGGTGAGGTGTACCCCAAA |
| HSL | TATCCGCTCTCCGGTTGA | CGAGCACTGGAGGAGTGTTT |
| IL-1b | ACCTATGTCTTGCCCGTGGA | AGGTCGTCATCATCCCACGA |
| IL-1R | GAGACCGACAAATGCACGGA | CTCCATCTTGGCGGGAACAA |
| IL-6 | AGCCACTGCCTTCCCTACTT | GCCATTGCACAACTCTTTTCTCA |
| IL-6R | AAGCAGGTCCAGCCACAATGTAG | CCAACTGACTTTGAGCCAACGAG |
| MC2R | GCTTTTGATCCCTGCTTTGAGTG | CATCTGTTAAAGAAGGAAAGGCTGG |
| MHC II | AGCACTGGGAGTTTGAAGAG | AAGCCATCACCTCCTGGTAT |
| MRAP | CCTCCCGGTGTGTGGCCTCT | GGGGACTATGCCTTACCTGTGGGG |
| NFKB1a | CACCAACTACAACGGCCACA | GCTCCTGAGCGTTGACATCA |
| NLRP3 | AGAAGCTGGGGTTGGTGAATT | GTTGTCTAACTCCAGCATCTG |
| Nur77 | GCGGAACCGCTGCCAGTTCT | GCATCTGGGGGCTGCTTGGG |
| PDE8B | ATGATGGGCTACCACAAAGGG | ATCCTGTGGATCTGCTTGCTAC |
| Per1 | CCTCGATGTAACGGCTTGTGT | GTCCGAGTGGCCAGGATCT |
| Per2 | CACCCTGAAAAGAAAGTGCGA | CAACGCCAAGGAGCTCAAGT |
| POMC | AGAGCTGGCTTTCCGCGACA | GGGCCCCTGAGCGACTGTAG |
| REV-ERBa | ACAGCTGACACCACCCAGATC | CATGGGCATAGGTGAAGATTTCT |
| SF-1 | CGCCAGGAGTTTGTCTGTCT | ACCTCCACCAGGCACAATAG |
| SIK1 | ACCAGCAGAGGCTGCTCCAGT | GGCGTTGGCAGCAGTGGGAT |
| StAR | CTGGCAGGCATGGCCACACA | GGCAGCCACCCCTTGAGGTC |
| TLR2 | TGGAGGTCTCCAGGTCAAATC | ACAGAGATGCCTGGGCAGAAT |
| TLR4 | TTTACAGAGGGGCAACCGCT | TTAGGAAGTACCTCTATGCAGGGAT |
| TNFa | AAATGGGCTCCCTCTATCAGTTC | TCTGCTTGGTGGTTTGCTACGAC |
| TNFaR | ATCCTGTCTCCAGTACCCCC | GTCTGCAGTGTCAAGCCGTT |
| TSPO | CTGAACTGGGCATGGCCCCC | GCCAAGCCAGGGTAGTGGCG |
|  |  |  |
|  |  |  |
|  |  |  |
