# Supplementary material for: Prolonged treatment with the synthetic glucocorticoid methylprednisolone affects adrenal steroidogenic function and response to inflammatory stress in the rat
Source: Brain Behav Immun. 2020 Jul;87:703–14. doi: 10.1016/j.bbi.2020.03.001 (PMC7327516; doi:10.1016/j.bbi.2020.03.001)
Supplement: Supplementary data 4 [file mmc4.docx]

**Supplementary Table 2: Effect of MPRED treatment and recovery on corticosterone secretion and pulse characteristics.**

|  |  | **Experimental group** | | |
| --- | --- | --- | --- | --- |
|  |  | **Ctrl** | **MP-T** | **MP-W** |
| **Mean corticosterone ^(a)^** | | 46.3±4.9 | 1.4±0.5*** | 32.3±3.9* |
| **Max corticosterone ^(a)^** | | 238.5±19.3 | 14.9±5.6*** | 167.3±25.3* |
| **AUC ^(a)^** | | 1112.7±114.0 | 21.3±9.5*** | 777.7±92.6* |
| **Baseline ^(b)^** | | 8.9±2.4 | 0.8±0.2** | 9.9±2.5^^^ |
| **Number of pulses ^(b)^** | | 17.3±1.3 | 0.7±0.4**** | 12.3±0.8 |
| **Pulse amplitude ^(c)^** | | 111.3±7.1 | nd | 63.6±12.5*** |
| **Pulse lengh ^(c)^** | | 0.71±0.03 | nd | 1.03±0.08* |
| **Pulse area ^(c)^** | | 47.8±4 | nd | 41.7±8.4 |
| **Inter-pulse interval ^(d)^** | | 1.3±0.1 | nd | 1.7±0.1* |
| **Pulse frequency ^(d)^** | | 0.72±0.05 | nd | 0.51±0.03*** |

Mean ± SEM of corticosterone secretion parameters and pulse characteristics analysed using the PULSAR algorithm. Data were calculated from 24-h corticosterone profiles from untreated rats (control group, Ctrl; n=7), rats treated with methylprednisolone (MP) in the drinking water (1g/L) for 5 days (MP treatment group, MPT; n=6), and rats treated with MP for 5 days and then left to recover for 5 days (MP recovery group, MPR; n=7). Data from MP-T rats were not included in the analysis of pulse characteristics as the number of pulses in these rats was low to non-detectable (nd). Blood samples were collected using and automated blood sampling system (data shown in Fig. 1B and Suppl. Fig. 2). AUC: area under curve. *P<0.05 **P<0.01, ***P<0.005, ****P<0.0001 vs Ctrl; ^^^^^P≤0.005 vs MPT. **^(a)^**One-way ANOVA followed by Tukey post-hoc test; **^(b)^** independent-samples Kruskal-Wallis Test pairwise comparisons with Bonferroni correction; ^(c)^ independent-samples Student t-test; **^(d)^**independent-samples Mann-Whitney U test).
