# Supplementary material for: Prolonged treatment with the synthetic glucocorticoid methylprednisolone affects adrenal steroidogenic function and response to inflammatory stress in the rat
Source: Brain Behav Immun. 2020 Jul;87:703–14. doi: 10.1016/j.bbi.2020.03.001 (PMC7327516; doi:10.1016/j.bbi.2020.03.001)
Supplement: Supplementary data 5 [file mmc5.docx]

**Supplementary Table 3: statistics results.**

|  | **Treatment** | | **Time** | | **Treatment x Time** | |
| --- | --- | --- | --- | --- | --- | --- |
| **Fig. 2** | **F(2,32)** | **P** | **F(1,32)** | **P** | **F(2,32)** | **P** |
| ACTH | 15.78 | **<0.00001** | 8.74 | **0.006** | 7.04 | **0.003** |
| A CORT | 24.97 | **<0.00001** | 36.37 | **<0.00001** | 17.43 | **<0.00001** |
| Adrenal weight | 18.45 | **<0.00001** | 0.14 | 0.714 | 1.80 | 0.185 |
| MC2R mRNA | 0.87 | 0.430 | 2.91 | 0.099 | 0.59 | 0.562 |
| MRAP mRNA | 29.28 | **<0.00001** | 38.04 | **<0.00001** | 16.05 | **<0.00001** |
| HSL mRNA | 20.73 | **<0.00001** | 4.24 | **0.049** | 4.79 | **0.017** |
| STAR mRNA | 34.52 | **<0.00001** | 33.46 | **<0.00001** | 15.68 | **<0.00001** |
| TSPO mRNA | 23.10 | **<0.00001** | 0.13 | 0.719 | 3.13 | 0.060 |
| CYP11a1 mRNA | 64.16 | **<0.00001** | 0.21 | 0.648 | 10.46 | **<0.00001** |
| HSD3b1 mRNA | 20.79 | **<0.00001** | 7.02 | **0.013** | 4.02 | **0.030** |
| CYP21a1 mRNA | 32.04 | **<0.00001** | 3.24 | 0.083 | 2.13 | 0.139 |
| CYP11b1 mRNA | 39.87 | **<0.00001** | 0.16 | 0.692 | 0.34 | 0.719 |
| HSD11b1 mRNA | 2.51 | 0.100 | 0.004 | 0.953 | 4.91 | **0.015** |
| PDE8b mRNA | 24.44 | **<0.00001** | 3.94 | 0.057 | 2.37 | 0.112 |
| HSL | 9.51 | **0.001** | 0.74 | 0.397 | 3.09 | 0.062 |
| STAR | 13.77 | **<0.00001** | 7.36 | **0.011** | 0.11 | 0.897 |
| **Fig. 3** | **F(2,32)** | **P** | **F(1,32)** | **P** | **F(2,32)** | **P** |
| CREB mRNA | 2.82 | 0.077 | 0.38 | 0.541 | 2.34 | 0.115 |
| NUR77 mRNA | 12.56 | **<0.00001** | 32.88 | **<0.00001** | 10.35 | **<0.00001** |
| SF1 mRNA | 14.85 | **<0.00001** | 14.58 | **0.001** | 4.89 | **0.015** |
| DAX1 mRNA | 6.28 | **0.006** | 2.92 | 0.099 | 0.21 | 0.809 |
| CRTC1 mRNA | 4.03 | **0.029** | 3.18 | 0.086 | 2.72 | 0.084 |
| CRTC2 mRNA | 8.30 | **0.002** | 0.11 | 0.741 | 3.68 | **0.039** |
| CRTC3 mRNA | 3.34 | **0.050** | 27.78 | **<0.00001** | 0.86 | 0.433 |
| SIK1 mRNA | 5.63 | **0.009** | 16.54 | **<0.00001** | 5.11 | **0.013** |
| **Fig. 4** | **F(2,32)** | **P** | **F(1,32)** | **P** | **F(2,32)** | **P** |
| BMAL mRNA | 2.00 | 0.155 | 47.38 | **<0.00001** | 5.85 | **0.008** |
| CLOCK mRNA | 4.10 | **0.028** | 0.03 | 0.868 | 0.87 | 0.432 |
| Per1 mRNA | 4.38 | **0.023** | 18.98 | **<0.00001** | 4.53 | **0.020** |
| Per2 mRNA | 9.80 | **0.001** | 57.87 | **<0.00001** | 12.87 | **<0.00001** |
| Cry1 mRNA | 23.31 | **<0.00001** | 62.98 | **<0.00001** | 24.04 | **<0.00001** |
| Cry2 mRNA | 10.12 | **0.001** | 9.39 | **0.005** | 4.41 | **0.022** |
| REV-ERBα mRNA | 11.94 | **<0.00001** | 33.69 | **<0.00001** | 3.08 | 0.062 |
| DBP mRNA | 14.95 | **<0.00001** | 16.01 | **<0.00001** | 5.93 | **0.007** |

**Supplementary Table 3 (continued)**

|  | **LPS** | | **Treatment** | | **LPS x Treatment** | |
| --- | --- | --- | --- | --- | --- | --- |
| **Fig. 5** | **F(2,34)** | **P** | **F 1,34)** | **P** | **F(2,34)** | **P** |
| ACTH | 150.99 | **<0.00001** | 34.08 | **<0.00001** | 31.42 | **<0.00001** |
| P CORT | 361.93 | **<0.00001** | 136.74 | **<0.00001** | 112.27 | **<0.00001** |
| A CORT | 154.12 | **<0.00001** | 56.99 | **<0.00001** | 47.26 | **<0.00001** |
| StAR mRNA | 2.39 | 0.133 | 61.79 | **<0.00001** | 4.70 | **0.017** |
| IL-1β mRNA (adrenal) | 111.05 | **<0.00001** | 8.79 | **0.001** | 8.84 | **0.001** |
| IL-6 mRNA (adrenal) | 49.65 | **<0.00001** | 2.33 | 0.115 | 2.33 | 0.115 |
| TNFα mRNA (adrenal) | 212.09 | **<0.00001** | 66.62 | **<0.00001** | 67.67 | **<0.00001** |
| MCH II mRNA (adrenal) | 1.90 | 0.179 | 8.52 | **0.001** | 0.26 | 0.775 |
|  | **F(2,30)** | **P** | **F 1,30)** | **P** | **F(2,30)** | **P** |
| IL-1β mRNA (liver) | 114.22 | **<0.00001** | 4.84 | **0.017** | 4.67 | **0.019** |
| IL-6 mRNA (liver) | 54.29 | **<0.00001** | 5.46 | **0.011** | 5.45 | **0.011** |
| TNFα mRNA (liver) | 66.82 | **<0.00001** | 1.25 | 0.304 | 1.20 | 0.319 |
| MCH II mRNA (liver) | 2.26 | 0.146 | 6.12 | **0.007** | 0.63 | 0.542 |
|  | **Treatment** | | **Time** | | **Treatment x Time** | |
| **Fig. 6** | **F(2,32)** | **P** | **F 1,32)** | **P** | **F(2,32)** | **P** |
| TLR2 mRNA | 3.43 | **0.047** | 13.97 | **0.001** | 0.96 | 0.396 |
| TLR4 mRNA | 2.01 | 0.154 | 0.05 | 0.825 | 0.60 | 0.557 |
| ANXA1 mRNA | 17.07 | **<0.00001** | 5.27 | **0.030** | 6.20 | **0.006** |
| FPR2 mRNA | 12.77 | **<0.00001** | 0.01 | 0.921 | 3.84 | **0.034** |
| NLRP3 mRNA | 0.96 | 0.396 | 0.00 | 0.974 | 0.15 | 0.861 |
| CASP1 mRNA | 0.91 | 0.413 | 3.05 | 0.092 | 3.86 | **0.034** |
| NFKB1A mRNA | 5.12 | **0.013** | 3.00 | 0.095 | 0.89 | 0.424 |
| IL-1R mRNA | 2.76 | 0.081 | 0.59 | 0.450 | 2.56 | 0.096 |
| IL-6R mRNA | 8.83 | **0.001** | 2.53 | 0.123 | 1.02 | 0.373 |
| TNFαR mRNA | 14.21 | **<0.00001** | 6.37 | **0.018** | 4.98 | **0.014** |
| **Suppl. Fig. 3** | **F(2,34)** | **P** | **F(1,34)** | **P** | **F(2,34)** | **P** |
| CRH | 0.34 | 0.715 | 15.80 | **<0.00001** | 0.03 | 0.968 |
| AVP | 0.04 | 0.960 | 32.89 | **<0.00001** | 0.14 | 0.872 |
| Hyp GR | 4.25 | **0.024** | 8.27 | **0.007** | 1.33 | 0.282 |
| Hyp MR | 1.65 | 0.210 | 10.06 | **0.004** | 2.14 | 0.136 |
|  | **F(2,33)** | **P** | **F(1,33)** | **P** | **F(2,33)** | **P** |
| POMC mRNA | 1.12 | 0.340 | 29.18 | **<0.00001** | 0.83 | 0.447 |
| CRHR1 mRNA | 1.44 | 0.255 | 130.59 | **<0.00001** | 0.30 | 0.744 |
| Pit GR mRNA | 3.23 | 0.055 | 41.52 | **<0.00001** | 1.10 | 0.347 |
| Pit MP mRNA | 0.22 | 0.807 | 60.91 | **<0.00001** | 1.38 | 0.269 |
| **Suppl. Fig. 4** | **F(2,32)** | **P** | **F(1,32)** | **P** | **F(2,32)** | **P** |
| HSD11b1 mRNA | 3.89 | 0.059 | 0.27 | 0.770 | 2.61 | 0.092 |

**Supplementary Table 3 (continued)**

|  | **Treatment** | | **Time** | | **Treatment x Time** | |
| --- | --- | --- | --- | --- | --- | --- |
| **Suppl. Fig. 5** | **F(2,32)** | **P** | **F(1,32)** | **P** | **F(2,32)** | **P** |
| BMAL mRNA | 2.41 | 0.109 | 191.77 | **<0.00001** | 10.62 | **<0.00001** |
| CLOCK mRNA | 0.28 | 0.762 | 22.60 | **<0.00001** | 3.78 | **0.036** |
| Per1 mRNA | 12.73 | **<0.00001** | 14.05 | **0.001** | 7.60 | **0.003** |
| Per2 mRNA | 3.69 | **0.039** | 45.50 | **<0.00001** | 4.95 | **0.015** |
| Cry1 mRNA | 2.69 | 0.087 | 5.40 | **0.028** | 1.40 | 0.264 |
| Cry2 mRNA | 2.50 | 0.102 | 28.98 | **<0.00001** | 1.98 | 0.158 |
| REV-ERBα mRNA | 2.06 | 0.147 | 52.96 | **<0.00001** | 1.71 | 0.201 |
| DBP mRNA | 2.03 | 0.151 | 47.88 | **<0.00001** | 2.39 | 0.112 |
| **Suppl. Fig. 6** | **F(2,32)** | **P** | **F(1,32)** | **P** | **F(2,32)** | **P** |
| TLR2 mRNA | 17.60 | **<0.00001** | 1.19 | 0.286 | 1.53 | 0.235 |
| TLR4 mRNA | 9.13 | **0.001** | 4.57 | **0.042** | 1.94 | 0.164 |
| ANXA1 mRNA | 1.97 | 0.159 | 0.07 | 0.788 | 0.42 | 0.662 |
| FPR2 mRNA | 0.29 | 0.754 | 17.54 | **<0.00001** | 0.47 | 0.629 |
| NLRP3 mRNA | 18.08 | **<0.00001** | 0.20 | 0.657 | 0.93 | 0.406 |
| CASP1 mRNA | 8.60 | **0.001** | 2.40 | 0.133 | 0.05 | 0.952 |
| NFKB1A mRNA | 10.26 | **0.001** | 2.77 | 0.108 | 0.19 | 0.828 |
| IL-1R mRNA | 8.30 | **0.002** | 9.91 | **0.004** | 1.53 | 0.235 |
| IL-6R mRNA | 4.31 | **0.024** | 16.45 | **<0.00001** | 4.53 | **0.021** |
| TNFαR mRNA | 3.45 | **0.047** | 1.64 | 0.211 | 1.34 | 0.280 |
| **Suppl. Fig. 7** | **F(2,32)** | **P** | **F(1,32)** | **P** | **F(2,32)** | **P** |
| CBG mRNA | 47.21 | **<0.00001** | 1.40 | 0.2470 | 0.48 | 0.624 |
| **Suppl. Fig. 8** | **F(2,32)** | **P** | **F(1,32)** | **P** | **F(2,32)** | **P** |
| HSL mRNA | 5.51 | **0.010** | 0.02 | 0.903 | 0.21 | 0.816 |
| STAR mRNA | 3.12 | 0.061 | 6.42 | **0.018** | 1.87 | 0.174 |
| TSPO mRNA | 10.84 | **<0.00001** | 0.13 | 0.725 | 1.04 | 0.368 |
| **Suppl. Fig. 9** | **F(2,32)** | **P** | **F(1,32)** | **P** | **F(2,32)** | **P** |
| GR mRNA (adrenal) | 1.09 | 0.350 | 0.00 | 0.978 | 1.43 | 0.256 |
| GILZ mRNA (adrenal) | 5.00 | **0.014** | 0.62 | 0.437 | 0.06 | 0.947 |
| FKBP5 mRNA (adrenal) | 1.20 | 0.317 | 15.33 | **0.001** | 4.85 | **0.016** |
| GR mRNA (liver) | 12.12 | **<0.00001** | 1.49 | 0.233 | 3.93 | **0.032** |
| GILZ mRNA (liver) | 5.81 | **0.008** | 3.48 | 0.073 | 0.34 | 0.712 |
| FKBP5 mRNA (liver) | 12.53 | **<0.00001** | 1.21 | 0.282 | 0.73 | 0.494 |
|  |  |  |  |  |  |  |
